# Trends and Disparities in Acute Myocardial Infarction‐Related Mortality With Co‐Listed Nicotine Dependence in the United States, 1999–2020

**DOI:** 10.1002/clc.70392

**Published:** 2026-06-23

**Authors:** Asad Ali Ahmed Cheema, Abu Huraira Bin Gulzar, Mishal Zehra, Ayesha Ahmed Cheema, Ayesha Saleem, Sayed Mohammad Saud Jalal, Nimra Shafi, Mirza Ammar Arshad, Kinza Raza, Iffat Ambreen Magsi

**Affiliations:** ^1^ Department of Medicine, International School of Medicine International University Kyrgyzstan Bishkek Kyrgyzstan; ^2^ Department of Medicine Services Institute of Medical Sciences Lahore Pakistan; ^3^ Department of Medicine Dow University of Health Sciences Karachi Pakistan; ^4^ Department of Medicine Al Tibri Medical College Karachi Karachi Sindh Pakistan; ^5^ Dow Medical College Dow University of Health Sciences Karachi Sindh Pakistan; ^6^ Department of Medicine Islam Medical College Sialkot Pakistan; ^7^ Department of Internal Medicine Arnot Ogden Medical Center Elmira New York USA; ^8^ Department of Medicine Rahbar Medical & Dental College Lahore Pakistan; ^9^ Department of Medicine Bakhtawar Amin Medical & Dental College Multan Pakistan; ^10^ Department of Medicine Shaheed Mohtarma Benazir Bhutto Medical University Karachi Pakistan

**Keywords:** acute myocardial infarction, adults, nicotine dependence, United States

## Abstract

**Background:**

Nicotine use is a major modifiable risk factor for acute myocardial infarction (AMI), yet national mortality patterns involving co‐listed nicotine dependence remain under‐characterized.

**Methods:**

Using CDC WONDER Multiple Cause of Death data from 1999 to 2020, we evaluated AMI‐related mortality among US adults aged ≥ 25 years with co‐listed death‐certificate‐coded nicotine dependence, operationally defined using ICD‐10 F17.0–F17.9. AMI was identified using ICD‐10 I21.0–I22.9. Age‐adjusted mortality rates (AAMRs) per 100 000 population were calculated using the 2000 US standard population. Temporal trends were assessed using Joinpoint regression and stratified by sex, age, race/ethnicity, region, urbanization, state, and place of death.

**Results:**

From 1999 to 2020, 357 167 AMI‐related deaths with co‐listed nicotine dependence occurred among adults aged ≥ 25 years. The AAMR increased from 1.64 to 9.46 per 100 000 population (average annual percent change, 10.27%; *p* < 0.001). Men had higher AAMRs than women (11.30 vs. 4.27), with significant increases in both groups. Mortality increased with age, highest among adults aged ≥ 85 years (33.55) and lowest among those aged 35–44 years (0.83). Non‐Hispanic American Indian/Alaska Native adults had the highest AAMR (10.30). Rates were higher in the Midwest and South, nonmetropolitan areas exceeded metropolitan areas, and the highest state‐level AAMRs occurred in North Dakota and Wyoming. Most deaths occurred in inpatient facilities or at home.

**Conclusion:**

AMI‐related mortality with co‐listed nicotine dependence increased substantially from 1999 to 2020, with persistent demographic and geographic disparities. These findings may inform targeted tobacco‐control, nicotine‐cessation, and cardiovascular prevention strategies for high‐risk populations over time.

## 1 Introduction

Nicotine dependence is one of the major public health concerns and accounts for a wide range of adverse outcomes, including cardiovascular diseases. According to the WHO report, Tobacco smoking is the leading cause of avoidable risk factors of cardiovascular diseases [1]. Approximately 23.6 million Americans have a nicotine use disorder, as reported by the National Institute on Drug Abuse [2]. In 2019, tobacco smoking was responsible for 7.7 million deaths and 200 million disability adjusted life‐years (DALYs), representing 20% male mortality [3].

Smoking significantly increases the risk of acute coronary and cerebrovascular events, including myocardial infarction, stroke, and sudden death [4]. Nicotine inflicts profound pathogenic changes, including accelerated atherogenesis involving major arteries including coronary arteries, carotids, and cerebral arteries, aorta, and peripheral circulation [5]. Smoking and nicotine dependence lead to exacerbation of angina pectoris and intermittent claudication, and result in restenosis and vasospastic angina post‐revascularization of coronary or peripheral arteries [5]. Moreover, nicotine impairs wound healing and alters inflammatory pathways [6] and triggers fibrosis with collagen deposition [7]. It also influences cardiovascular physiology and drives cellular and molecular transformation into cardiovascular and pulmonary tissues [8].

As compared with nonsmokers, acute myocardial infarction (AMI) is linked with larger thrombus load with less severe atherosclerosis in people who smoke [5]. This study aims to explore the relationship between nicotine dependence and MI mortality using data from the CDC WONDER database, which provides comprehensive mortality and population health information. We will also explore disparities in MI mortality related to nicotine dependence across demographic groups including age, sex, race, and socioeconomic status. The findings will help guide policy decisions, clinical interventions, and public health campaigns to address nicotine addiction and reduce the burden of cardiovascular disease in at‐risk populations.

## 2 Methods

### 2.1 Study Setting and Population

Mortality data were obtained from the Centers for Disease Control and Prevention's Wide‐ranging Online Data for Epidemiologic Research (CDC WONDER) Multiple Cause of Death database from 1999 to 2020. This database contains death‐certificate‐derived mortality data from all 50 US states and the District of Columbia and has been widely used for national mortality trend analyses. The study population included US adults aged ≥ 25 years with AMI‐related mortality and co‐listed nicotine dependence. AMI‐related deaths were identified using ICD‐10 codes I21.0, I21.1, I21.2, I21.3, I21.4, I21.9, I22.0, I22.1, I22.8, and I22.9. Nicotine dependence was operationally defined using the full ICD‐10 F17.0–F17.9 code block for mental and behavioral disorders due to tobacco use, including F17.0, F17.1, F17.2, F17.3, F17.4, F17.5, F17.6, F17.7, F17.8, and F17.9. This definition was selected because F17.0–F17.9 represents the ICD‐10 tobacco/nicotine‐related mental and behavioral disorder code block available in CDC WONDER multiple‐cause‐of‐death data and is consistent with prior CDC WONDER tobacco‐related mortality analyses. Therefore, throughout this manuscript, “nicotine dependence” refers to death‐certificate‐coded F17.0–F17.9 tobacco/nicotine‐related mental and behavioral disorders rather than self‐reported smoking status, current smoking, former smoking, pack‐year history, vaping exposure, secondhand smoke exposure, or any tobacco exposure. Codes for tobacco history, tobacco counseling, nonspecific tobacco exposure, and toxic effects of tobacco/nicotine were not included because the objective was to evaluate AMI‐related mortality with co‐listed nicotine dependence as a coded disorder, not all tobacco exposure or nicotine poisoning. Because this study used publicly available, deidentified mortality data, institutional review board approval and informed consent were not required. The study was reported according to the Strengthening the Reporting of Observational Studies in Epidemiology (STROBE) guidelines.

### 2.2 Data Abstraction

Our data for demographic variables were based on population size, age group distribution, gender, place of death, urban‐rural classification, region, and racial/ethnic background were abstracted. Race and ethnicity were categorized as Non‐Hispanic (NH) White, NH Black/African American, NH Asian, NH American Indian/Alaskan Native, and Hispanic (Latino). This classification is based on the reported data on death certificates and aligns with the previously performed analysis of the CDC WONDER database [9]. The National Center for Health Statistics Urban‐Rural Classification Scheme was employed to stratify the population geographically by urban (large metropolitan area [population > 1 million], medium/small metropolitan area [population between 50 000–999 999]) and rural (population < 50 000) counties per the 2013 US census classification [10]. Furthermore, we divided the United States into Northeast, Midwest, South, and West regions according to the US Census Bureau [11].

### 2.3 Statistical Analysis

We evaluated gender, race, age, urbanization, geographical region, and census data‐related trends by calculating both crude and age‐adjusted mortality rates (AAMR) per 100 000 individuals. The 2000 US population was administered as a reference point for AAMR standardization.

To examine the changes over time in mortality rates, we employed the Joinpoint Regression Program (Version 5.0.2, National Cancer Institute). The temporal trends in AAMR were evaluated by fitting log‐linear regression models to crude data patterns to calculate the annual percentage change (APC) in AAMR with its 95% confidence interval (CI). An APC was classified as increasing or decreasing if the slope explains the deviation in mortality was markedly different from 0, using a two‐tailed *t*‐test with statistical significance of *p* < 0.05.

## 3 Results

### 3.1 Overall Mortality Trends

**Figure 1:**
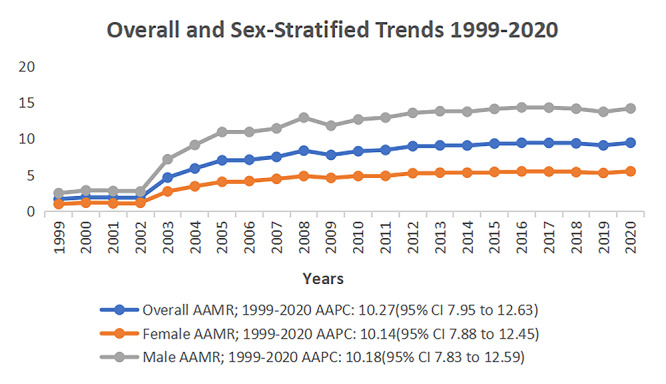
The graph demonstrating overall annual trends of age‐adjusted mortality rates (AAMR) across both sexes from 1999 to 2020.

Between 1999 and 2020, 357 167 AMI‐related deaths with co‐listed nicotine dependence were recorded among US adults aged ≥ 25 years. The overall AAMR was 7.36 per 100 000 population (95% CI, 7.34–7.39). The AAMR increased from 1.64 per 100 000 population in 1999 to 9.46 per 100 000 population in 2020. Joinpoint analysis identified an initial significant increase from 1999 to 2005 (APC, 34.42%; 95% CI, 24.56%–45.07%), followed by a slower but significant increase from 2005 to 2020 (APC, 1.87%; 95% CI, 0.88%–2.88%). Across the full study period, the average annual percent change (AAPC) was 10.27% (95% CI, 7.95%–12.64%) (Figure 1, Table 1, Supporting Information S1: Table S1).

**Table 1:** Annual percent change (APC) in age‐adjusted mortality rates (1999–2020) by different variables, highlighting temporal trends across key segments.

Cohort	Segment	Lower endpoint	Upper endpoint	APC (95% CI)	Overall AAPC (95% CI)
Overall cohort	1	1999	2005	34.4239* (24.5632 to 45.0652)	10.2704* (7.9523 to 12.6383)
Age (years)					
35–44	1	1999	2005	22.4660* (15.4195 to 29.9426)	7.7039* (5.9176 to 9.5204)
	2	2005	2020	2.3100* (1.4373 to 3.1902)	
45–54	1	1999	2005	24.6839* (17.7577 to 32.0176)	8.1933* (6.1012 to 10.3267)
	2	2005	2012	4.8653* (1.3767 to 8.4739)	
	3	2012	2020	−0.0311* (−2.0881 to 2.0692)	
55–64	1	1999	2005	25.7198* (17.5386 to 34.4705)	9.0203* (6.9944 to 11.0846)
65–74	1	1999	2005	32.5344* (23.3494 to 42.4034)	9.6519* (7.4791 to 11.8686)
	2	2005	2020	1.6462* (0.7164 to 2.5846)	
75–84	1	1999	2005	41.6767* (29.5794 to 54.9034)	11.4222* (8.6901 to 14.2231)
85+	1	1999	2005	55.7769* (39.2374 to 74.2811)	14.5420* (11.0856 to 18.106)
Gender					
Female	1	1999	2005	34.0838* (24.493 to 44.4135)	10.1455* (7.8873 to 12.4509)
	2	2005	2020	1.8129* (0.8343 to 2.801)	
Male	1	1999	2005	34.7401* (24.6562 to 45.6398)	10.1872* (7.8305 to 12.5955)
	2	2005	2020	1.6682* (0.69 to 2.6558)	
Race/ethnicity					
NH American Indian or Alaska Native	1	1999	2005	35.6776* (21.9944 to 50.8954)	9.6758* (6.4875 to 12.9596)
	2	2005	2020	0.7285 (−0.5707 to 2.0445)	
NH Asian or Pacific Islander	1	1999	2007	25.5528* (19.5153 to 31.8953)	8.8829* (6.8929 to 10.9099)
	2	2007	2020	−0.2558 (−1.2919 to 0.7912)	
NH Black or African American	1	1999	2005	31.3919* (22.0296 to 41.4725)	10.5693* (8.3184 to 12.867)
	2	2005	2020	3.1956* (2.2405 to 4.1596)	
NH White	1	1999	2005	33.5322* (24.9973 to 42.6498)	10.3797* (8.3403 to 12.4575)
	2	2005	2020	2.2847* (1.309 to 3.2698)	
Hispanic or Latino	1	1999	2004	44.4990* (22.6704 to 70.2119)	9.4325* (5.4188 to 13.5991)
	2	2004	2020	0.3279 (−0.9642 to 1.6369)	
Census region					
Northeast	1	1999	2005	51.4242* (38.1789 to 65.9391)	11.8725* (9.0728 to 14.7441)
	2	2005	2020	−0.8861 (−1.9581 to 0.1976)	
Midwest	1	1999	2008	26.2212* (20.8436 to 31.838)	10.9808* (8.9123 to 13.0885)
	2	2008	2020	0.7707 (−0.6081 to 2.1686)	
South	1	1999	2004	32.7595* (17.7104 to 49.7326)	9.6605* (6.6662 to 12.739)
	2	2004	2020	3.3019* (2.1932 to 4.4225)	
West	1	1999	2001	−2.9964 (−21.6863 to 20.1539)	5.7295* (2.6378 to 8.9144)
	2	2001	2004	48.3295* (24.4294 to 76.8202)	
Urbanization					
Metropolitan	1	1999	2005	35.5531* (25.7916 to 46.072)	10.1674* (7.8932 to 12.4895)
	2	2005	2020	1.3982* (0.4173 to 2.3888)	
Non‐metropolitan	1	1999	2004	33.7138* (21.5308 to 47.1182)	10.5256* (7.8615 to 13.2555)
	2	2004	2013	6.6287* (3.9402 to 9.3868)	
	3	2013	2020	1.024 (−1.5787 to 3.6955)	

* Indicates that the Annual Percent Change (APC) or Average Annual Percent Change (AAPC) is statistically significantly different from zero (*p* < 0.05).

### 3.2 Mortality by Gender

Men accounted for 242 847 deaths (67.99%), whereas women accounted for 114 320 deaths (32.01%). Men had a higher overall AAMR than women (11.30 vs. 4.27 per 100 000 population). In men, the AAMR increased from 2.47 in 1999 to 14.21 in 2020, with a significant overall AAPC of 10.19% (95% CI, 7.83%–12.60%). In women, the AAMR increased from 0.96 in 1999 to 5.49 in 2020, with a significant overall AAPC of 10.15% (95% CI, 7.89%–12.45%). Both sexes showed a significant early increase from 1999 to 2005, followed by slower significant increases from 2005 to 2020 (Figure 1, Table 1, Supporting Information S1: Table S2).

### 3.3 Mortality Stratified by Age Group

Age‐specific crude mortality rates (CMRs) increased with advancing age. The lowest overall CMR was observed among adults aged 25–34 years (0.10 per 100 000 population), followed by adults aged 35–44 years (0.83 per 100 000 population). The highest overall CMR was observed among adults aged ≥ 85 years (33.55 per 100 000 population), followed by adults aged 75–84 years (28.40 per 100 000 population), 65–74 years (19.63 per 100 000 population), 55–64 years (11.18 per 100 000 population), and 45–54 years (4.06 per 100 000 population). The largest number of deaths occurred among adults aged 65–74 years (100 184 deaths; 28.05%), followed by 55–64 years (85 710 deaths; 24.00%) and 75–84 years (84 788 deaths; 23.74%).

Among modeled age strata, all age groups demonstrated significant positive AAPCs across the study period. The largest overall increase was observed among adults aged ≥ 85 years (AAPC, 14.54%; 95% CI, 11.09%–18.11%), followed by adults aged 75–84 years (AAPC, 11.42%; 95% CI, 8.69%–14.22%), 65–74 years (AAPC, 9.65%; 95% CI, 7.48%–11.87%), 55–64 years (AAPC, 9.02%; 95% CI, 6.99%–11.08%), 45–54 years (AAPC, 8.19%; 95% CI, 6.10%–10.33%), and 35–44 years (AAPC, 7.70%; 95% CI, 5.92%–9.52%). After the early increase, the 45–54‐year age group plateaued from 2012 to 2020 (APC, −0.03%; 95% CI, −2.09% to 2.07%) (Table 1, Supporting Information S1: Table S3).

### 3.4 Mortality Stratified by Race and Ethnicity

When stratified by race and ethnicity, the highest overall AAMR was observed among NH American Indian or Alaska Native adults (10.30 per 100 000 population), followed by NH White adults (8.21 per 100 000 population), NH Black or African American adults (6.27 per 100 000 population), Hispanic or Latino adults (3.33 per 100 000 population), and NH Asian or Pacific Islander adults (1.73 per 100 000 population). Across the full study period, significant positive AAPCs were observed among NH Black or African American adults (AAPC, 10.57%; 95% CI, 8.32%–12.87%), NH White adults (AAPC, 10.38%; 95% CI, 8.34%–12.46%), NH American Indian or Alaska Native adults (AAPC, 9.68%; 95% CI, 6.49%–12.96%), Hispanic or Latino adults (AAPC, 9.43%; 95% CI, 5.42%–13.60%), and NH Asian or Pacific Islander adults (AAPC, 8.88%; 95% CI, 6.89%–10.91%).

**Figure 2:**
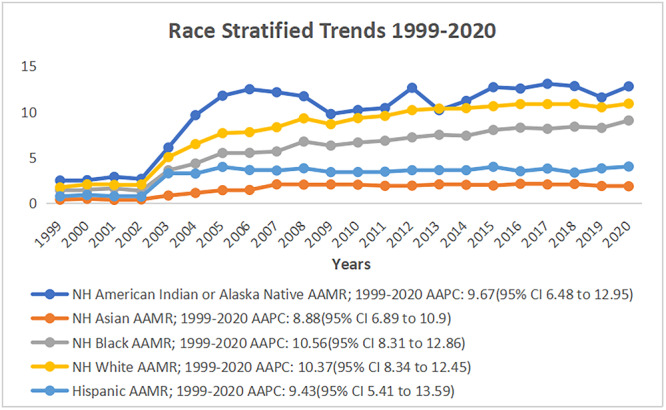
The graph demonstrating stratification of annual trends of AAMR across various races/ethnicities from 1999 to 2020.

Joinpoint patterns varied by group. NH Black or African American and NH White adults showed significant increases during both the early and later segments. NH American Indian or Alaska Native adults showed a significant increase from 1999 to 2005, followed by a stable pattern from 2005 to 2020. NH Asian or Pacific Islander adults showed a significant increase from 1999 to 2007, followed by a stable pattern from 2007 to 2020. Hispanic or Latino adults showed a significant increase from 1999 to 2004, followed by a stable pattern from 2004 to 2020 (Figure 2, Table 1, Supporting Information S1: Table S4).

### 3.5 Mortality Stratified by US Census Region

Regional differences were observed across the study period. The Midwest had the highest overall AAMR (9.67 per 100 000 population), followed by the South (7.50 per 100 000 population), Northeast (7.09 per 100 000 population), and West (5.03 per 100 000 population). In 2020, AAMRs were highest in the Midwest (12.86 per 100 000 population), followed by the South (10.41 per 100 000 population), Northeast (8.28 per 100 000 population), and West (5.60 per 100 000 population).

**Figure 3:**
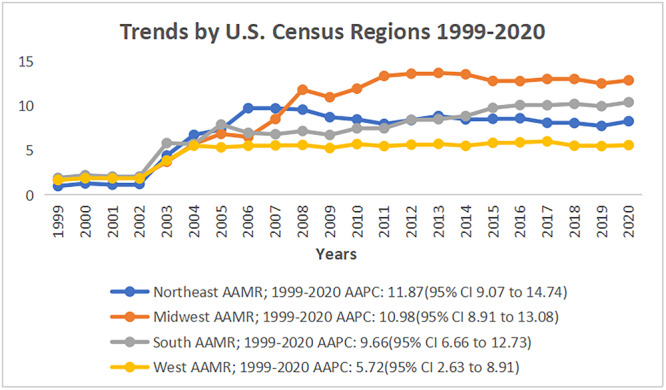
The graph demonstrating overall annual trends of AAMR across various regions from 1999 to 2020.

All US Census regions demonstrated significant positive AAPCs over the full study period. The Northeast had the largest AAPC (11.87%; 95% CI, 9.07%–14.74%), followed by the Midwest (10.98%; 95% CI, 8.91%–13.09%), South (9.66%; 95% CI, 6.67%–12.74%), and West (5.73%; 95% CI, 2.64%–8.91%). Segment‐specific trends showed a significant early increase in the Northeast from 1999 to 2005, followed by a stable pattern from 2005 to 2020. The Midwest increased significantly from 1999 to 2008 and then plateaued from 2008 to 2020. The South increased significantly from 1999 to 2004 and continued to increase significantly from 2004 to 2020. The West was stable from 1999 to 2001, increased significantly from 2001 to 2004, and then plateaued from 2004 to 2020 (Figure 3, Table 1, Supporting Information S1: Table S5).

### 3.6 State‐Level Variation

**Figure 4:**
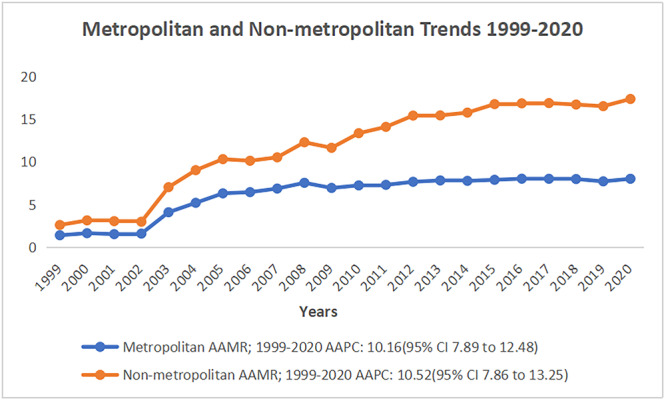
Choropleth map demonstrating annual trends of AAMR across various states from 1999 to 2020.

State‐level AAMRs varied widely across the United States. The highest overall AAMRs were observed in North Dakota (19.59 per 100 000 population), Wyoming (18.63 per 100 000 population), Idaho (17.37 per 100 000 population), South Dakota (16.09 per 100 000 population), and Vermont (14.46 per 100 000 population). The lowest AAMRs were observed in California (1.29 per 100 000 population), Massachusetts (2.39 per 100 000 population), Alabama (2.65 per 100 000 population), the District of Columbia (3.41 per 100 000 population), and Nevada (3.60 per 100 000 population) (Figure 4, Supporting Information S1: Table S6).

### 3.7 Mortality Stratified by Urbanization

Nonmetropolitan areas had higher AMI‐related mortality with co‐listed nicotine dependence than metropolitan areas. The overall AAMR was 12.09 per 100 000 population in nonmetropolitan areas compared with 6.43 per 100 000 population in metropolitan areas. In nonmetropolitan areas, the AAMR increased from 2.61 in 1999 to 17.34 in 2020, with a significant overall AAPC of 10.53% (95% CI, 7.86%–13.26%). In metropolitan areas, the AAMR increased from 1.41 in 1999 to 8.01 in 2020, with a significant overall AAPC of 10.17% (95% CI, 7.89%–12.49%).

**Figure 5:**
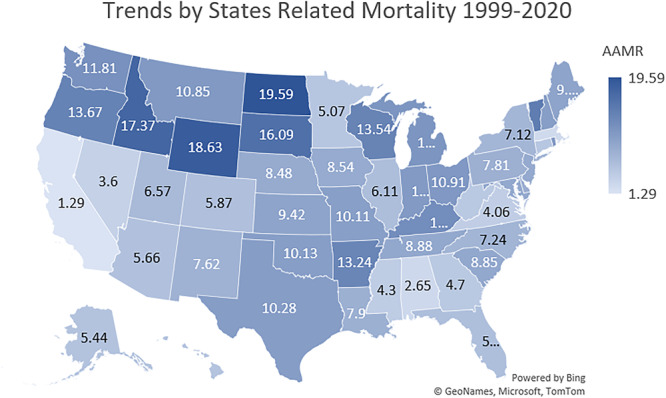
The graph demonstrating annual trends of AAMR across various regions from 1999 to 2020.

Joinpoint analysis showed that metropolitan areas increased significantly from 1999 to 2005 and continued to increase, albeit more slowly, from 2005 to 2020. Nonmetropolitan areas increased significantly from 1999 to 2004 and from 2004 to 2013, followed by a stable pattern from 2013 to 2020 (Figure 5, Table 1, Supporting Information S1: Table S7).

### 3.8 Place of Death

Most deaths occurred in inpatient medical facilities (132 203 deaths; 37.01%), followed by the decedent's home (117 419 deaths; 32.88%) and outpatient or emergency department medical facilities (64 347 deaths; 18.02%). Other locations included nursing home or long‐term care facilities (21 333 deaths; 5.97%), other places of death (11 283 deaths; 3.16%), hospice facilities (5560 deaths; 1.56%), dead on arrival at a medical facility (4602 deaths; 1.29%), place of death unknown (362 deaths; 0.10%), and medical facility status unknown (58 deaths; 0.02%).

## 4 Discussion

In this national CDC WONDER analysis of 357 167 AMI‐related deaths with co‐listed nicotine dependence from 1999 to 2020, we observed a substantial overall increase in AAMR. The overall AAMR increased from 1.64 to 9.46 per 100 000 population, with a significant positive AAPC across the study period. Men had consistently higher AAMRs than women, and both sexes showed significant overall increases. Age‐specific CMRs were highest among adults aged ≥ 85 years and lowest among adults aged 35–44 years. Racial and ethnic differences were observed, with the highest overall AAMR among NH American Indian or Alaska Native adults. Regionally, overall AAMRs were highest in the Midwest and South, and nonmetropolitan areas had higher AAMRs than metropolitan areas. State‐level AAMRs varied widely, with the highest overall rates observed in North Dakota and Wyoming. Most deaths occurred in inpatient medical facilities or at home. Selected later Joinpoint segments with nonsignificant negative or near‐null APC were interpreted as stable or plateaued segment‐specific patterns and not as evidence of an overall declining mortality trend (Central Illustration 1).

**Central Illustration 1:**
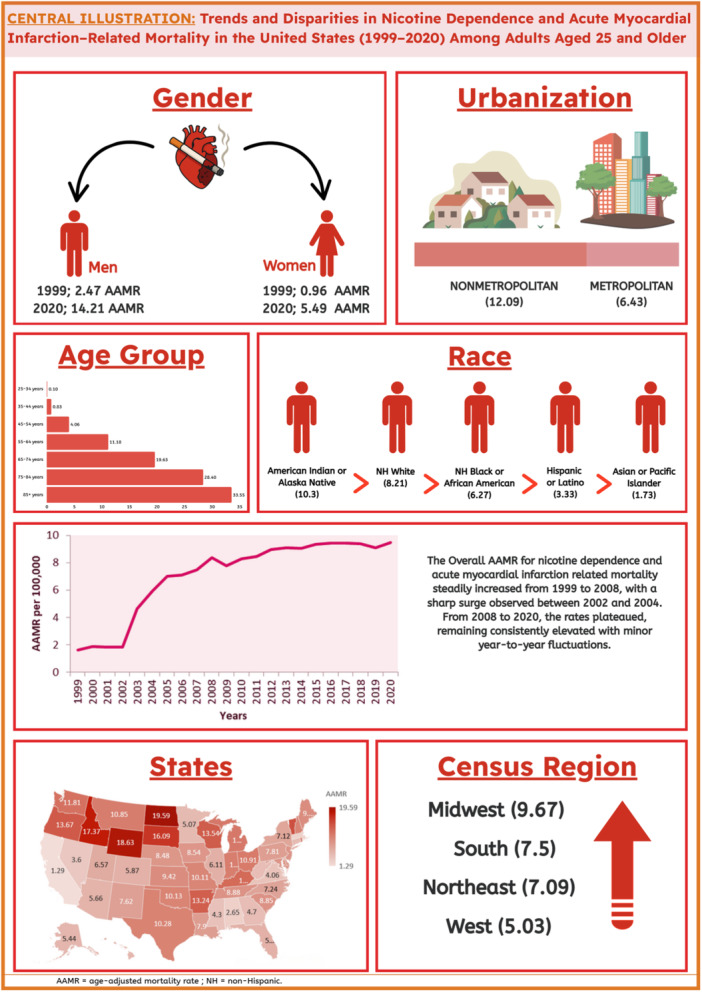
National CDC WONDER data showed AMI‐related mortality with co‐listed nicotine dependence increased substantially overall from 1999 to 2020, with higher burden among men, older adults, non‐Hispanic American Indian/Alaska Native adults, nonmetropolitan areas, and Midwest/South regions.

The observed increase in AAMR should be interpreted in the context of the established cardiovascular harms of tobacco/nicotine exposure and changing nicotine‐product use patterns over recent decades, including cigarette smoking and the emergence of vaping and e‐cigarette use [12, 13]. Nicotine is a well‐known risk factor for cardiovascular disease [5, 12, 13]. It exerts its adverse effects on the cardiovascular system through hemodynamic changes, leading to abrupt changes in heart rate and blood pressure that result in increased myocardial oxygen demand. It also leads to a pro‐inflammatory and pro‐thrombotic state, by resulting in neutrophil activation, endothelial injury, dyslipidemia, and prostacyclin inhibition, all contributing to increased platelet aggregation and acceleration of atherosclerosis, and thus resulting in increased morbidity and mortality [5]. Nicotine use, in the form of cigarette smoking, contributes to larger adverse effects, including AMI; however, nicotine consumption through vaping, e‐cigarettes, and nicotine replacement therapy is not free of the harmful effects and can contribute to adverse outcomes with long‐term use [5, 13, 14].

A significant gender disparity in AAMR was observed in our study, with males being affected nearly three times more than females. Despite global trends indicating rising nicotine use among females, notable differences in consumption patterns persist [15]. Males generally exhibit higher rates of smoking and nicotine dependence, along with a greater propensity for developing nicotine use disorder compared to females [16]. Additionally, male gender is considered a risk factor for cardiovascular disease due to hormonal differences, with estrogen offering a protective effect to females prior to menopause. These differences combined account for an increased mortality related to nicotine dependence and AMI among males [17]. To address this, targeted interventions such as public awareness campaigns, education, and counseling are essential to reduce nicotine dependence, particularly among adolescents, who are especially vulnerable to initiating and sustaining these habits.

Our study demonstrated a higher mortality rate among older adults, aligning with prior evidence that suggests an increased prevalence of nicotine dependence in this population [18, 19]. Several factors may contribute to this trend. Older adults often have longer cumulative exposure to nicotine, leading to more adverse cardiovascular events. Moreover, it is challenging to introduce lifestyle modifications in this patient population due to deeply ingrained habits, a lower perceived benefit of cessation, or a reduced willingness to adapt to behavioral changes. In addition, older adults may be less exposed to public health messaging about the dangers of nicotine, especially when compared to younger individuals who are often the focus of prevention campaigns [18, 19]. This lack of awareness, coupled with potential gaps in counseling or cessation support, may further exacerbate nicotine dependence in this group. To address this disparity, we need to develop age‐specific strategies to reduce nicotine dependence among older adults. Tailored education, improved access to cessation resources, and increased engagement by healthcare providers can play a pivotal role in addressing this issue.

Considerable demographic and geographic differences were observed. The highest AAMRs were observed among NH American Indian or Alaska Native adults, followed by NH White adults, and regional rates were highest in the Midwest and South. Nonmetropolitan areas also had higher AAMRs than metropolitan areas. Prior studies have linked lower socioeconomic status and lower educational attainment with higher nicotine dependence and cardiovascular mortality; however, the present CDC WONDER analysis does not include individual‐level socioeconomic, clinical, or healthcare‐access data. Therefore, these demographic and geographic findings should be interpreted as descriptive population‐level patterns rather than evidence of specific causal pathways [20].

This study has several limitations. First, CDC WONDER mortality data are derived from death certificates and ICD‐10 coding; therefore, AMI and nicotine dependence may be misclassified, incompletely documented, or underreported. Death certificate data also depend on the accuracy and completeness of certifier documentation and cannot confirm clinical adjudication of AMI or nicotine dependence. Second, nicotine dependence was operationally defined using death‐certificate‐coded ICD‐10 F17.0–F17.9 codes. This approach captures coded tobacco/nicotine‐related mental and behavioral disorders but does not identify all individuals with active smoking, former smoking, vaping, smokeless tobacco use, secondhand smoke exposure, pack‐year history, nicotine product type, duration of use, intensity of use, or cessation treatment. Because nicotine dependence may be underreported on death certificates, the observed mortality burden likely reflects deaths with documented F17.0–F17.9‐coded nicotine dependence rather than the full burden of AMI mortality associated with tobacco exposure. Third, CDC WONDER does not provide patient‐level clinical data, including comorbidities, laboratory findings, angiographic findings, AMI severity, treatment received, medication use, revascularization status, or cause‐specific clinical pathways preceding death. Fourth, the serial cross‐sectional design describes population‐level mortality trends and cannot establish causality, temporality, individual‐level risk, or whether nicotine dependence independently contributed to AMI‐related mortality. Fifth, demographic and geographic analyses are ecological and should not be interpreted as identifying individual‐level determinants of mortality. Race and ethnicity are based on death certificate reporting, and urban‐rural classification is assigned at the county level rather than the individual level. Sixth, data on healthcare access, treatment delays, socioeconomic factors, insurance status, local tobacco‐control policies, nicotine‐cessation resources, and regional healthcare infrastructure were not available, limiting interpretation of observed demographic, regional, and urban‐rural differences.

## 5 Conclusion

In this national analysis, AMI‐related mortality with co‐listed nicotine dependence increased substantially from 1999 to 2020. The burden was higher among men, older adults, NH American Indian or Alaska Native adults, nonmetropolitan areas, and selected Midwestern and Southern regions. These findings highlight persistent demographic and geographic disparities and may inform targeted tobacco‐control, nicotine‐cessation, and cardiovascular prevention strategies for high‐risk populations to help reduce AMI‐related mortality among adults with death‐certificate‐coded nicotine dependence.

## Author Contributions

**Asad Ali Ahmed Cheema:** investigation, methodology, conceptualization, visualization, writing – original draft, writing – review and editing. **Abu Huraira Bin Gulzar:** conceptualization, methodology, investigation, validation, writing – review and editing, writing – original draft. **Mishal Zehra:** writing – original draft, writing – review and editing, investigation. **Ayesha Ahmed Cheema:** writing – original draft, writing – review and editing, formal analysis. **Ayesha Saleem:** writing – original draft, writing – review and editing. **Syed Mohammad Saud Jalal:** formal analysis, writing – review and editing. **Nimra Shafi:** writing – original draft. **Mirza Ammar Arshad:** writing – review and editing. **Kinza Raza:** illustration. **Iffat Ambreen Magsi:** writing – review and editing.

## Funding

The authors have nothing to report.

## Ethics Statement

The authors have nothing to report.

## Consent

The authors have nothing to report.

## Conflicts of Interest

The authors declare no conflicts of interest.

## Supporting information


**Table S1:** Overall annual AMI‐related mortality with co‐listed nicotine dependence.
**Table S2:** Sex‐specific annual mortality.
**Table S3:** Age group‐specific annual crude mortality.
**Table S4:** Race/ethnicity‐specific annual mortality.
**Table S5:** U.S. Census region‐specific annual mortality.
**Table S6:** State‐level total mortality, ranked by AAMR.
**Table S7:** Urbanization‐specific annual mortality.

## Data Availability

The data used in this study are publicly available from the Centers for Disease Control and Prevention's Wide‐ranging Online Data for Epidemiologic Research (CDC WONDER) Multiple Cause of Death database. The database can be accessed at https://wonder.cdc.gov/mcd.html. The extracted data supporting the findings of this study are included in the article and Supplementary Material.
