# Correction to “Trends and Disparities in Acute Myocardial Infarction‐Related Mortality With Co‐Listed Nicotine Dependence in the United States, 1999–2020”

**DOI:** 10.1002/clc.70432

**Published:** 2026-08-10

Cheema AAA, Bin Gulzar AH, Zehra M, et al. Trends and disparities in acute myocardial infarction‐related mortality with co‐listed nicotine dependence in the United States, 1999–2020. *Clinical Cardiology* 49, no. 6 (2026): e70392, https://doi.org/10.1002/clc.70392.

In the published version of this article, the captions for Figures 1, 4, and 5 were incorrect. Also, Table 1 had a number of inconsistencies and missing rows of data. The correct figure captions and Table 1 are as follows:

**Table 1:** Annual percentage change (APC) and overall average annual percentage change (AAPC) in age‐adjusted mortality rates per 100,000 (1999 to 2020) by overall and other demographic variables, highlighting temporal trends across key segments.

Cohort	Segment	Lower endpoint	Upper endpoint	APC (95% CI)	Overall AAPC (95% CI)
Overall cohort	1	1999	2005	34.4239* (24.5632 to 45.0652)	10.2704* (7.9523 to 12.6383)
	2	2005	2020	1.8713* (0.8752 to 2.8772)	
**Age (Years)**					
35–44 years	1	1999	2005	22.4660* (15.4195 to 29.9426)	7.7039* (5.9176 to 9.5204)
	2	2005	2020	2.3100* (1.4373 to 3.1902)	
45–54 years	1	1999	2005	24.6839* (17.7577 to 32.0176)	8.1933* (6.1012 to 10.3267)
	2	2005	2012	4.8653* (1.3767 to 8.4739)	
	3	2012	2020	−0.0311 (−2.0881 to 2.0692)	
55–64 years	1	1999	2005	25.7198* (17.5386 to 34.4705)	9.0203* (6.9944 to 11.0846)
	2	2005	2020	2.9791* (2.0931 to 3.8728)	
65–74 years	1	1999	2005	32.5344* (23.3494 to 42.4034)	9.6519* (7.4791 to 11.8686)
	2	2005	2020	1.6462* (0.7164 to 2.5846)	
75–84 years	1	1999	2005	41.6767* (29.5794 to 54.9034)	11.4222* (8.6901 to 14.2231)
	2	2005	2020	1.2141* (0.0878 to 2.3532)	
85+ years	1	1999	2005	55.7769* (39.2374 to 74.2811)	14.5420* (11.0856 to 18.106)
	2	2005	2020	1.2860* (0.199 to 2.3849)	
**Gender**					
Female	1	1999	2005	34.0838* (24.493 to 44.4135)	10.1455* (7.8873 to 12.4509)
	2	2005	2020	1.8129* (0.8343 to 2.801)	
Male	1	1999	2005	34.7401* (24.6562 to 45.6398)	10.1872* (7.8305 to 12.5955)
	2	2005	2020	1.6682* (0.69 to 2.6558)	
**Race/Ethnicity**					
NH American Indian or Alaska Native	1	1999	2005	35.6776* (21.9944 to 50.8954)	9.6758* (6.4875 to 12.9596)
	2	2005	2020	0.7285 (−0.5707 to 2.0445)	
NH Asian or Pacific Islander	1	1999	2007	25.5528* (19.5153 to 31.8953)	8.8829* (6.8929 to 10.9099)
	2	2007	2020	−0.2558 (−1.2919 to 0.7912)	
NH Black or African American	1	1999	2005	31.3919* (22.0296 to 41.4725)	10.5693* (8.3184 to 12.867)
	2	2005	2020	3.1956* (2.2405 to 4.1596)	
NH White	1	1999	2005	33.5322* (24.9973 to 42.6498)	10.3797* (8.3403 to 12.4575)
	2	2005	2020	2.2847* (1.309 to 3.2698)	
Hispanic or Latino	1	1999	2004	44.4990* (22.6704 to 70.2119)	9.4325* (5.4188 to 13.5991)
	2	2004	2020	0.3279 (−0.9642 to 1.6369)	
**Census region**					
Northeast	1	1999	2005	51.4242* (38.1789 to 65.9391)	11.8725* (9.0728 to 14.7441)
	2	2005	2020	−0.8861 (−1.9581 to 0.1976)	
Midwest	1	1999	2008	26.2212* (20.8436 to 31.838)	10.9808* (8.9123 to 13.0885)
	2	2008	2020	0.7707 (−0.6081 to 2.1686)	
South	1	1999	2004	32.7595* (17.7104 to 49.7326)	9.6605* (6.6662 to 12.739)
	2	2004	2020	3.3019* (2.1932 to 4.4225)	
West	1	1999	2001	−2.9964 (−21.6863 to 20.1539)	5.7295* (2.6378 to 8.9144)
	2	2001	2004	48.3295* (24.4294 to 76.8202)	
	3	2004	2020	0.3007 (−0.0993 to 0.7023)	
**Urbanization**					
Metropolitan	1	1999	2005	35.5531* (25.7916 to 46.072)	10.1674* (7.8932 to 12.4895)
	2	2005	2020	1.3982* (0.4173 to 2.3888)	
Non‐metropolitan	1	1999	2004	33.7138* (21.5308 to 47.1182)	10.5256* (7.8615 to 13.2555)
	2	2004	2013	6.6287* (3.9402 to 9.3868)	
	3	2013	2020	1.024 (−1.5787 to 3.6955)	

* *P*‐value was significant < 0.05.

**Figure 1.** The graph demonstrating overall and gender‐specific annual trends of Age‐Adjusted Mortality Rates (AAMR) from 1999 to 2020.

**Figure 4.** The graph demonstrating annual trends of AAMR across metropolitan and non‐metropolitan areas from 1999 to 2020.

**Figure 5.** Choropleth map demonstrating annual trends of AAMR across various states from 1999 to 2020.

