# Supplementary material for: Trends and Disparities in Acute Myocardial Infarction‐Related Mortality With Co‐Listed Nicotine Dependence in the United States, 1999–2020
Source: Clin Cardiol. 2026 Jun 23;49(6):e70392. doi: 10.1002/clc.70392 (PMC13288362; doi:10.1002/clc.70392)
Supplement: Supplementary file 1 — Table S1: Overall annual AMI‐related mortality with co‐listed nicotine dependence. Table S2: Sex‐specific annual mortality. Table S3: Age group‐specific annual crude mortality. Table S4: Race/ethnicity‐specific annual mortality. Table S5: U.S. Census region‐specific annual mortality. Table S6: State‐level total mortality, ranked by AAMR. Table S7: Urbanization‐specific annual mortality. [file CLC-49-e70392-s001.docx]

**Supplemental Material**

**Trends and Disparities in Acute Myocardial Infarction-Related Mortality with Co-Listed Nicotine Dependence in the United States, 1999-2020**

**Abbreviations:** AAMR, age-adjusted mortality rate; AMI, acute myocardial infarction; CI, confidence interval; CMR, crude mortality rate; CDC WONDER, Centers for Disease Control and Prevention Wide-ranging Online Data for Epidemiologic Research.

**Supplemental table order**

Supplemental Table S1. Overall annual AMI-related mortality with co-listed nicotine dependence.

Supplemental Table S2. Sex-specific annual mortality.

Supplemental Table S3. Age group-specific annual crude mortality.

Supplemental Table S4. Race/ethnicity-specific annual mortality.

Supplemental Table S5. U.S. Census region-specific annual mortality.

Supplemental Table S6. State-level total mortality, ranked by AAMR.

Supplemental Table S7. Urbanization-specific annual mortality.

## **Supplemental Table S1. Overall annual AMI-related mortality with co-listed nicotine dependence, 1999-2020.**

| **Year** | **Deaths** | **Population** | **AAMR (95% CI)** | **% total deaths** |
| --- | --- | --- | --- | --- |
| 1999 | 2,901 | 180,408,769 | 1.64 (1.58-1.70) | 0.81 |
| 2000 | 3,440 | 181,984,640 | 1.89 (1.83-1.96) | 0.96 |
| 2001 | 3,365 | 184,305,128 | 1.84 (1.78-1.90) | 0.94 |
| 2002 | 3,449 | 186,208,028 | 1.85 (1.79-1.91) | 0.97 |
| 2003 | 8,813 | 188,090,429 | 4.63 (4.54-4.73) | 2.47 |
| 2004 | 11,354 | 190,205,384 | 5.89 (5.78-5.99) | 3.18 |
| 2005 | 13,809 | 192,551,384 | 7.02 (6.91-7.14) | 3.87 |
| 2006 | 14,226 | 195,019,359 | 7.10 (6.98-7.22) | 3.98 |
| 2007 | 15,381 | 197,403,777 | 7.49 (7.37-7.60) | 4.31 |
| 2008 | 17,530 | 199,795,090 | 8.36 (8.24-8.49) | 4.91 |
| 2009 | 16,594 | 202,107,016 | 7.76 (7.64-7.88) | 4.65 |
| 2010 | 18,036 | 203,891,983 | 8.28 (8.16-8.41) | 5.05 |
| 2011 | 18,865 | 206,592,936 | 8.45 (8.33-8.57) | 5.28 |
| 2012 | 20,514 | 208,826,037 | 8.98 (8.86-9.11) | 5.74 |
| 2013 | 21,346 | 211,085,314 | 9.09 (8.97-9.22) | 5.98 |
| 2014 | 21,832 | 213,809,280 | 9.07 (8.95-9.19) | 6.11 |
| 2015 | 22,942 | 216,553,817 | 9.34 (9.21-9.46) | 6.42 |
| 2016 | 23,792 | 218,641,417 | 9.45 (9.32-9.57) | 6.66 |
| 2017 | 24,303 | 221,447,331 | 9.43 (9.31-9.55) | 6.80 |
| 2018 | 24,569 | 223,311,190 | 9.37 (9.25-9.49) | 6.88 |
| 2019 | 24,362 | 224,981,167 | 9.09 (8.97-9.21) | 6.82 |
| 2020 | 25,744 | 226,635,013 | 9.46 (9.34-9.58) | 7.21 |
| **Total** | **357,167** | **4,473,854,489** | **7.36 (7.34-7.39)** | **100.00** |

## **Supplemental Table S2. Sex-specific annual AMI-related mortality with co-listed nicotine dependence, 1999-2020.**

| **Stratum** | **Year** | **Deaths** | **Population** | **AAMR (95% CI)** | **% total deaths** |
| --- | --- | --- | --- | --- | --- |
| Male | 1999 | 1,952 | 86,285,677 | 2.47 (2.36-2.58) | 0.55 |
| Male | 2000 | 2,299 | 87,120,538 | 2.85 (2.74-2.97) | 0.64 |
| Male | 2001 | 2,274 | 88,320,720 | 2.76 (2.65-2.88) | 0.64 |
| Male | 2002 | 2,272 | 89,280,325 | 2.70 (2.59-2.81) | 0.64 |
| Male | 2003 | 5,913 | 90,197,132 | 7.16 (6.98-7.35) | 1.66 |
| Male | 2004 | 7,649 | 91,283,860 | 9.15 (8.94-9.36) | 2.14 |
| Male | 2005 | 9,335 | 92,453,545 | 10.94 (10.71-11.16) | 2.61 |
| Male | 2006 | 9,558 | 93,690,917 | 10.95 (10.72-11.17) | 2.68 |
| Male | 2007 | 10,300 | 94,889,947 | 11.44 (11.21-11.66) | 2.88 |
| Male | 2008 | 11,883 | 96,106,934 | 12.94 (12.70-13.18) | 3.33 |
| Male | 2009 | 11,157 | 97,272,830 | 11.82 (11.60-12.05) | 3.12 |
| Male | 2010 | 12,241 | 98,174,557 | 12.68 (12.45-12.91) | 3.43 |
| Male | 2011 | 12,859 | 99,588,456 | 12.94 (12.71-13.16) | 3.60 |
| Male | 2012 | 13,964 | 100,737,208 | 13.59 (13.36-13.82) | 3.91 |
| Male | 2013 | 14,562 | 101,912,532 | 13.82 (13.59-14.05) | 4.08 |
| Male | 2014 | 14,841 | 103,227,661 | 13.75 (13.52-13.98) | 4.16 |
| Male | 2015 | 15,658 | 104,606,455 | 14.14 (13.92-14.37) | 4.38 |
| Male | 2016 | 16,320 | 105,650,504 | 14.35 (14.13-14.58) | 4.57 |
| Male | 2017 | 16,651 | 107,089,305 | 14.32 (14.10-14.54) | 4.66 |
| Male | 2018 | 16,843 | 108,045,192 | 14.17 (13.95-14.39) | 4.72 |
| Male | 2019 | 16,713 | 108,896,735 | 13.74 (13.53-13.96) | 4.68 |
| Male | 2020 | 17,603 | 109,725,881 | 14.21 (14.00-14.43) | 4.93 |
| **Male** | **Total** | **242,847** | **2,154,556,911** | **11.30 (11.25-11.34)** | **67.99** |
| Female | 1999 | 949 | 94,123,092 | 0.96 (0.90-1.02) | 0.27 |
| Female | 2000 | 1,141 | 94,864,102 | 1.14 (1.07-1.21) | 0.32 |
| Female | 2001 | 1,091 | 95,984,408 | 1.05 (0.99-1.11) | 0.31 |
| Female | 2002 | 1,177 | 96,927,703 | 1.13 (1.06-1.19) | 0.33 |
| Female | 2003 | 2,900 | 97,893,297 | 2.71 (2.61-2.81) | 0.81 |
| Female | 2004 | 3,705 | 98,921,524 | 3.42 (3.31-3.53) | 1.04 |
| Female | 2005 | 4,474 | 100,097,839 | 4.04 (3.92-4.16) | 1.25 |
| Female | 2006 | 4,668 | 101,328,442 | 4.15 (4.03-4.27) | 1.31 |
| Female | 2007 | 5,081 | 102,513,830 | 4.45 (4.33-4.58) | 1.42 |
| Female | 2008 | 5,647 | 103,688,156 | 4.84 (4.72-4.97) | 1.58 |
| Female | 2009 | 5,437 | 104,834,186 | 4.58 (4.46-4.70) | 1.52 |
| Female | 2010 | 5,795 | 105,717,426 | 4.84 (4.72-4.97) | 1.62 |
| Female | 2011 | 6,006 | 107,004,480 | 4.86 (4.73-4.98) | 1.68 |
| Female | 2012 | 6,550 | 108,088,829 | 5.23 (5.10-5.36) | 1.83 |
| Female | 2013 | 6,784 | 109,172,782 | 5.31 (5.18-5.44) | 1.90 |
| Female | 2014 | 6,991 | 110,581,619 | 5.31 (5.18-5.43) | 1.96 |
| Female | 2015 | 7,284 | 111,947,362 | 5.41 (5.28-5.54) | 2.04 |
| Female | 2016 | 7,472 | 112,990,913 | 5.48 (5.35-5.60) | 2.09 |
| Female | 2017 | 7,652 | 114,358,026 | 5.46 (5.34-5.58) | 2.14 |
| Female | 2018 | 7,726 | 115,265,998 | 5.38 (5.26-5.50) | 2.16 |
| Female | 2019 | 7,649 | 116,084,432 | 5.26 (5.14-5.38) | 2.14 |
| Female | 2020 | 8,141 | 116,909,132 | 5.49 (5.37-5.61) | 2.28 |
| **Female** | **Total** | **114,320** | **2,319,297,578** | **4.27 (4.24-4.29)** | **32.01** |

## **Supplemental Table S3. Age group-specific annual crude mortality for AMI-related deaths with co-listed nicotine dependence, 1999-2020.**

| **Age group** | **Year** | **Deaths** | **Population** | **CMR (95% CI)** | **% total deaths** |
| --- | --- | --- | --- | --- | --- |
| 25-34 years | 1999 | Suppressed | 40,178,406 | Suppressed | Suppressed |
| 25-34 years | 2000 | 15 | 39,891,724 | Unreliable (0.02-0.06) | 0.00 |
| 25-34 years | 2001 | 13 | 39,471,522 | Unreliable (0.02-0.06) | 0.00 |
| 25-34 years | 2002 | Suppressed | 39,349,646 | Suppressed | Suppressed |
| 25-34 years | 2003 | 26 | 39,243,795 | 0.07 (0.04-0.10) | 0.01 |
| 25-34 years | 2004 | 32 | 39,266,556 | 0.08 (0.06-0.12) | 0.01 |
| 25-34 years | 2005 | 27 | 39,258,647 | 0.07 (0.05-0.10) | 0.01 |
| 25-34 years | 2006 | 42 | 39,395,179 | 0.11 (0.08-0.14) | 0.01 |
| 25-34 years | 2007 | 31 | 39,713,463 | 0.08 (0.05-0.11) | 0.01 |
| 25-34 years | 2008 | 51 | 40,207,473 | 0.13 (0.09-0.17) | 0.01 |
| 25-34 years | 2009 | 41 | 40,723,342 | 0.10 (0.07-0.14) | 0.01 |
| 25-34 years | 2010 | 57 | 41,063,948 | 0.14 (0.11-0.18) | 0.02 |
| 25-34 years | 2011 | 65 | 41,790,498 | 0.16 (0.12-0.20) | 0.02 |
| 25-34 years | 2012 | 65 | 42,309,321 | 0.15 (0.12-0.20) | 0.02 |
| 25-34 years | 2013 | 62 | 42,844,587 | 0.14 (0.11-0.19) | 0.02 |
| 25-34 years | 2014 | 48 | 43,516,504 | 0.11 (0.08-0.15) | 0.01 |
| 25-34 years | 2015 | 54 | 44,137,202 | 0.12 (0.09-0.16) | 0.02 |
| 25-34 years | 2016 | 64 | 44,677,243 | 0.14 (0.11-0.18) | 0.02 |
| 25-34 years | 2017 | 65 | 45,342,672 | 0.14 (0.11-0.18) | 0.02 |
| 25-34 years | 2018 | 47 | 45,697,774 | 0.10 (0.08-0.14) | 0.01 |
| 25-34 years | 2019 | 70 | 45,940,321 | 0.15 (0.12-0.19) | 0.02 |
| 25-34 years | 2020 | 78 | 46,069,646 | 0.17 (0.13-0.21) | 0.02 |
| **25-34 years** | **Total** | **965** | **920,089,469** | **0.10 (0.10-0.11)** | **0.27** |
| 35-44 years | 1999 | 127 | 45,076,677 | 0.28 (0.23-0.33) | 0.04 |
| 35-44 years | 2000 | 149 | 45,148,527 | 0.33 (0.28-0.38) | 0.04 |
| 35-44 years | 2001 | 138 | 45,051,752 | 0.31 (0.26-0.36) | 0.04 |
| 35-44 years | 2002 | 158 | 44,640,649 | 0.35 (0.30-0.41) | 0.04 |
| 35-44 years | 2003 | 281 | 44,154,206 | 0.64 (0.56-0.71) | 0.08 |
| 35-44 years | 2004 | 297 | 43,800,275 | 0.68 (0.60-0.76) | 0.08 |
| 35-44 years | 2005 | 361 | 43,505,538 | 0.83 (0.74-0.92) | 0.10 |
| 35-44 years | 2006 | 376 | 43,243,801 | 0.87 (0.78-0.96) | 0.11 |
| 35-44 years | 2007 | 362 | 42,796,230 | 0.85 (0.76-0.93) | 0.10 |
| 35-44 years | 2008 | 351 | 42,192,486 | 0.83 (0.74-0.92) | 0.10 |
| 35-44 years | 2009 | 364 | 41,487,811 | 0.88 (0.79-0.97) | 0.10 |
| 35-44 years | 2010 | 373 | 41,070,606 | 0.91 (0.82-1.00) | 0.10 |
| 35-44 years | 2011 | 424 | 40,627,954 | 1.04 (0.94-1.14) | 0.12 |
| 35-44 years | 2012 | 435 | 40,516,420 | 1.07 (0.97-1.17) | 0.12 |
| 35-44 years | 2013 | 431 | 40,452,690 | 1.07 (0.96-1.17) | 0.12 |
| 35-44 years | 2014 | 404 | 40,513,133 | 1.00 (0.90-1.09) | 0.11 |
| 35-44 years | 2015 | 445 | 40,589,783 | 1.10 (0.99-1.20) | 0.12 |
| 35-44 years | 2016 | 399 | 40,470,156 | 0.99 (0.89-1.08) | 0.11 |
| 35-44 years | 2017 | 446 | 40,875,370 | 1.09 (0.99-1.19) | 0.12 |
| 35-44 years | 2018 | 458 | 41,277,888 | 1.11 (1.01-1.21) | 0.13 |
| 35-44 years | 2019 | 461 | 41,659,144 | 1.11 (1.01-1.21) | 0.13 |
| 35-44 years | 2020 | 501 | 42,136,192 | 1.19 (1.08-1.29) | 0.14 |
| **35-44 years** | **Total** | **7,741** | **931,287,288** | **0.83 (0.81-0.85)** | **2.17** |
| 45-54 years | 1999 | 440 | 36,577,819 | 1.20 (1.09-1.32) | 0.12 |
| 45-54 years | 2000 | 518 | 37,677,952 | 1.37 (1.26-1.49) | 0.15 |
| 45-54 years | 2001 | 540 | 39,386,268 | 1.37 (1.26-1.49) | 0.15 |
| 45-54 years | 2002 | 547 | 39,992,194 | 1.37 (1.25-1.48) | 0.15 |
| 45-54 years | 2003 | 991 | 40,819,954 | 2.43 (2.28-2.58) | 0.28 |
| 45-54 years | 2004 | 1,306 | 41,629,930 | 3.14 (2.97-3.31) | 0.37 |
| 45-54 years | 2005 | 1,594 | 42,495,904 | 3.75 (3.57-3.94) | 0.45 |
| 45-54 years | 2006 | 1,664 | 43,286,159 | 3.84 (3.66-4.03) | 0.47 |
| 45-54 years | 2007 | 1,819 | 43,939,939 | 4.14 (3.95-4.33) | 0.51 |
| 45-54 years | 2008 | 2,079 | 44,460,447 | 4.68 (4.48-4.88) | 0.58 |
| 45-54 years | 2009 | 1,941 | 44,867,088 | 4.33 (4.13-4.52) | 0.54 |
| 45-54 years | 2010 | 2,110 | 45,006,716 | 4.69 (4.49-4.89) | 0.59 |
| 45-54 years | 2011 | 2,236 | 44,718,203 | 5.00 (4.79-5.21) | 0.63 |
| 45-54 years | 2012 | 2,275 | 44,268,738 | 5.14 (4.93-5.35) | 0.64 |
| 45-54 years | 2013 | 2,295 | 43,767,532 | 5.24 (5.03-5.46) | 0.64 |
| 45-54 years | 2014 | 2,229 | 43,458,851 | 5.13 (4.92-5.34) | 0.62 |
| 45-54 years | 2015 | 2,272 | 43,188,161 | 5.26 (5.04-5.48) | 0.64 |
| 45-54 years | 2016 | 2,278 | 42,786,679 | 5.32 (5.11-5.54) | 0.64 |
| 45-54 years | 2017 | 2,186 | 42,374,952 | 5.16 (4.94-5.37) | 0.61 |
| 45-54 years | 2018 | 2,159 | 41,631,699 | 5.19 (4.97-5.40) | 0.60 |
| 45-54 years | 2019 | 2,016 | 40,874,902 | 4.93 (4.72-5.15) | 0.56 |
| 45-54 years | 2020 | 2,185 | 40,366,133 | 5.41 (5.19-5.64) | 0.61 |
| **45-54 years** | **Total** | **37,680** | **927,576,220** | **4.06 (4.02-4.10)** | **10.55** |
| 55-64 years | 1999 | 776 | 23,778,026 | 3.26 (3.03-3.49) | 0.22 |
| 55-64 years | 2000 | 856 | 24,274,684 | 3.53 (3.29-3.76) | 0.24 |
| 55-64 years | 2001 | 830 | 25,105,295 | 3.31 (3.08-3.53) | 0.23 |
| 55-64 years | 2002 | 887 | 26,703,332 | 3.32 (3.10-3.54) | 0.25 |
| 55-64 years | 2003 | 1,916 | 28,008,945 | 6.84 (6.53-7.15) | 0.54 |
| 55-64 years | 2004 | 2,410 | 29,305,304 | 8.22 (7.90-8.55) | 0.67 |
| 55-64 years | 2005 | 2,972 | 30,641,497 | 9.70 (9.35-10.05) | 0.83 |
| 55-64 years | 2006 | 3,052 | 31,930,113 | 9.56 (9.22-9.90) | 0.85 |
| 55-64 years | 2007 | 3,479 | 33,128,434 | 10.50 (10.15-10.85) | 0.97 |
| 55-64 years | 2008 | 3,955 | 34,157,063 | 11.58 (11.22-11.94) | 1.11 |
| 55-64 years | 2009 | 3,826 | 35,405,600 | 10.81 (10.46-11.15) | 1.07 |
| 55-64 years | 2010 | 4,264 | 36,482,729 | 11.69 (11.34-12.04) | 1.19 |
| 55-64 years | 2011 | 4,593 | 38,062,140 | 12.07 (11.72-12.42) | 1.29 |
| 55-64 years | 2012 | 4,997 | 38,586,202 | 12.95 (12.59-13.31) | 1.40 |
| 55-64 years | 2013 | 5,249 | 39,316,431 | 13.35 (12.99-13.71) | 1.47 |
| 55-64 years | 2014 | 5,368 | 40,077,581 | 13.39 (13.04-13.75) | 1.50 |
| 55-64 years | 2015 | 5,713 | 40,877,819 | 13.98 (13.61-14.34) | 1.60 |
| 55-64 years | 2016 | 5,961 | 41,463,144 | 14.38 (14.01-14.74) | 1.67 |
| 55-64 years | 2017 | 6,061 | 41,995,658 | 14.43 (14.07-14.80) | 1.70 |
| 55-64 years | 2018 | 6,007 | 42,272,636 | 14.21 (13.85-14.57) | 1.68 |
| 55-64 years | 2019 | 6,003 | 42,448,537 | 14.14 (13.78-14.50) | 1.68 |
| 55-64 years | 2020 | 6,535 | 42,403,677 | 15.41 (15.04-15.79) | 1.83 |
| **55-64 years** | **Total** | **85,710** | **766,424,847** | **11.18 (11.11-11.26)** | **24.00** |
| 65-74 years | 1999 | 828 | 18,418,909 | 4.50 (4.19-4.80) | 0.23 |
| 65-74 years | 2000 | 982 | 18,390,986 | 5.34 (5.01-5.67) | 0.27 |
| 65-74 years | 2001 | 933 | 18,384,179 | 5.08 (4.75-5.40) | 0.26 |
| 65-74 years | 2002 | 946 | 18,388,535 | 5.14 (4.82-5.47) | 0.26 |
| 65-74 years | 2003 | 2,374 | 18,500,915 | 12.83 (12.32-13.35) | 0.66 |
| 65-74 years | 2004 | 2,983 | 18,667,533 | 15.98 (15.41-16.55) | 0.84 |
| 65-74 years | 2005 | 3,558 | 18,881,697 | 18.84 (18.22-19.46) | 1.00 |
| 65-74 years | 2006 | 3,625 | 19,203,027 | 18.88 (18.26-19.49) | 1.01 |
| 65-74 years | 2007 | 3,883 | 19,698,727 | 19.71 (19.09-20.33) | 1.09 |
| 65-74 years | 2008 | 4,474 | 20,505,679 | 21.82 (21.18-22.46) | 1.25 |
| 65-74 years | 2009 | 4,375 | 21,233,099 | 20.60 (19.99-21.22) | 1.22 |
| 65-74 years | 2010 | 4,667 | 21,713,429 | 21.49 (20.88-22.11) | 1.31 |
| 65-74 years | 2011 | 4,882 | 22,481,738 | 21.72 (21.11-22.32) | 1.37 |
| 65-74 years | 2012 | 5,598 | 23,985,392 | 23.34 (22.73-23.95) | 1.57 |
| 65-74 years | 2013 | 5,907 | 25,216,766 | 23.42 (22.83-24.02) | 1.65 |
| 65-74 years | 2014 | 6,301 | 26,398,290 | 23.87 (23.28-24.46) | 1.76 |
| 65-74 years | 2015 | 6,626 | 27,550,517 | 24.05 (23.47-24.63) | 1.86 |
| 65-74 years | 2016 | 7,037 | 28,630,330 | 24.58 (24.00-25.15) | 1.97 |
| 65-74 years | 2017 | 7,427 | 29,683,446 | 25.02 (24.45-25.59) | 2.08 |
| 65-74 years | 2018 | 7,402 | 30,492,316 | 24.27 (23.72-24.83) | 2.07 |
| 65-74 years | 2019 | 7,406 | 31,483,433 | 23.52 (22.99-24.06) | 2.07 |
| 65-74 years | 2020 | 7,970 | 32,549,398 | 24.49 (23.95-25.02) | 2.23 |
| **65-74 years** | **Total** | **100,184** | **510,458,341** | **19.63 (19.50-19.75)** | **28.05** |
| 75-84 years | 1999 | 580 | 12,224,914 | 4.74 (4.36-5.13) | 0.16 |
| 75-84 years | 2000 | 759 | 12,361,180 | 6.14 (5.70-6.58) | 0.21 |
| 75-84 years | 2001 | 738 | 12,593,618 | 5.86 (5.44-6.28) | 0.21 |
| 75-84 years | 2002 | 709 | 12,764,864 | 5.55 (5.15-5.96) | 0.20 |
| 75-84 years | 2003 | 2,395 | 12,896,438 | 18.57 (17.83-19.31) | 0.67 |
| 75-84 years | 2004 | 3,145 | 12,989,903 | 24.21 (23.36-25.06) | 0.88 |
| 75-84 years | 2005 | 3,746 | 13,074,802 | 28.65 (27.73-29.57) | 1.05 |
| 75-84 years | 2006 | 3,867 | 13,095,151 | 29.53 (28.60-30.46) | 1.08 |
| 75-84 years | 2007 | 4,004 | 13,087,439 | 30.59 (29.65-31.54) | 1.12 |
| 75-84 years | 2008 | 4,554 | 13,076,102 | 34.83 (33.82-35.84) | 1.28 |
| 75-84 years | 2009 | 4,070 | 13,022,775 | 31.25 (30.29-32.21) | 1.14 |
| 75-84 years | 2010 | 4,369 | 13,061,122 | 33.45 (32.46-34.44) | 1.22 |
| 75-84 years | 2011 | 4,414 | 13,175,230 | 33.50 (32.51-34.49) | 1.24 |
| 75-84 years | 2012 | 4,833 | 13,272,634 | 36.41 (35.39-37.44) | 1.35 |
| 75-84 years | 2013 | 4,851 | 13,446,519 | 36.08 (35.06-37.09) | 1.36 |
| 75-84 years | 2014 | 4,900 | 13,682,690 | 35.81 (34.81-36.81) | 1.37 |
| 75-84 years | 2015 | 5,089 | 13,923,174 | 36.55 (35.55-37.55) | 1.42 |
| 75-84 years | 2016 | 5,301 | 14,233,534 | 37.24 (36.24-38.25) | 1.48 |
| 75-84 years | 2017 | 5,384 | 14,706,551 | 36.61 (35.63-37.59) | 1.51 |
| 75-84 years | 2018 | 5,664 | 15,394,374 | 36.79 (35.83-37.75) | 1.59 |
| 75-84 years | 2019 | 5,590 | 15,969,872 | 35.00 (34.09-35.92) | 1.57 |
| 75-84 years | 2020 | 5,826 | 16,451,547 | 35.41 (34.50-36.32) | 1.63 |
| **75-84 years** | **Total** | **84,788** | **298,504,433** | **28.40 (28.21-28.60)** | **23.74** |
| 85+ years | 1999 | 147 | 4,154,018 | 3.54 (2.97-4.11) | 0.04 |
| 85+ years | 2000 | 161 | 4,239,587 | 3.80 (3.21-4.38) | 0.05 |
| 85+ years | 2001 | 173 | 4,312,494 | 4.01 (3.41-4.61) | 0.05 |
| 85+ years | 2002 | 193 | 4,368,808 | 4.42 (3.79-5.04) | 0.05 |
| 85+ years | 2003 | 830 | 4,466,176 | 18.58 (17.32-19.85) | 0.23 |
| 85+ years | 2004 | 1,181 | 4,545,883 | 25.98 (24.50-27.46) | 0.33 |
| 85+ years | 2005 | 1,551 | 4,693,299 | 33.05 (31.40-34.69) | 0.43 |
| 85+ years | 2006 | 1,600 | 4,865,929 | 32.88 (31.27-34.49) | 0.45 |
| 85+ years | 2007 | 1,803 | 5,039,545 | 35.78 (34.13-37.43) | 0.50 |
| 85+ years | 2008 | 2,066 | 5,195,840 | 39.76 (38.05-41.48) | 0.58 |
| 85+ years | 2009 | 1,977 | 5,367,301 | 36.83 (35.21-38.46) | 0.55 |
| 85+ years | 2010 | 2,196 | 5,493,433 | 39.98 (38.30-41.65) | 0.61 |
| 85+ years | 2011 | 2,251 | 5,737,173 | 39.24 (37.61-40.86) | 0.63 |
| 85+ years | 2012 | 2,311 | 5,887,330 | 39.25 (37.65-40.85) | 0.65 |
| 85+ years | 2013 | 2,551 | 6,040,789 | 42.23 (40.59-43.87) | 0.71 |
| 85+ years | 2014 | 2,582 | 6,162,231 | 41.90 (40.28-43.52) | 0.72 |
| 85+ years | 2015 | 2,743 | 6,287,161 | 43.63 (42.00-45.26) | 0.77 |
| 85+ years | 2016 | 2,752 | 6,380,331 | 43.13 (41.52-44.74) | 0.77 |
| 85+ years | 2017 | 2,734 | 6,468,682 | 42.27 (40.68-43.85) | 0.77 |
| 85+ years | 2018 | 2,832 | 6,544,503 | 43.27 (41.68-44.87) | 0.79 |
| 85+ years | 2019 | 2,816 | 6,604,958 | 42.63 (41.06-44.21) | 0.79 |
| 85+ years | 2020 | 2,649 | 6,658,420 | 39.78 (38.27-41.30) | 0.74 |
| **85+ years** | **Total** | **40,099** | **119,513,891** | **33.55 (33.22-33.88)** | **11.23** |

## **Supplemental Table S4. Race/ethnicity-specific annual AMI-related mortality with co-listed nicotine dependence, 1999-2020.**

| **Stratum** | **Year** | **Deaths** | **Population** | **AAMR (95% CI)** | **% total deaths** |
| --- | --- | --- | --- | --- | --- |
| Non-Hispanic American Indian or Alaska Native | 1999 | 22 | 1,243,850 | 2.49 (1.52-3.84) | 0.01 |
| Non-Hispanic American Indian or Alaska Native | 2000 | 21 | 1,293,461 | 2.53 (1.53-3.96) | 0.01 |
| Non-Hispanic American Indian or Alaska Native | 2001 | 30 | 1,307,212 | 2.89 (1.90-4.20) | 0.01 |
| Non-Hispanic American Indian or Alaska Native | 2002 | 31 | 1,325,864 | 2.67 (1.78-3.87) | 0.01 |
| Non-Hispanic American Indian or Alaska Native | 2003 | 62 | 1,343,849 | 6.10 (4.60-7.94) | 0.02 |
| Non-Hispanic American Indian or Alaska Native | 2004 | 97 | 1,364,416 | 9.64 (7.69-11.94) | 0.03 |
| Non-Hispanic American Indian or Alaska Native | 2005 | 118 | 1,387,305 | 11.77 (9.50-14.05) | 0.03 |
| Non-Hispanic American Indian or Alaska Native | 2006 | 134 | 1,410,814 | 12.48 (10.21-14.74) | 0.04 |
| Non-Hispanic American Indian or Alaska Native | 2007 | 136 | 1,434,577 | 12.15 (9.94-14.36) | 0.04 |
| Non-Hispanic American Indian or Alaska Native | 2008 | 136 | 1,460,440 | 11.71 (9.59-13.82) | 0.04 |
| Non-Hispanic American Indian or Alaska Native | 2009 | 112 | 1,485,009 | 9.77 (7.81-11.73) | 0.03 |
| Non-Hispanic American Indian or Alaska Native | 2010 | 125 | 1,503,624 | 10.20 (8.29-12.12) | 0.04 |
| Non-Hispanic American Indian or Alaska Native | 2011 | 141 | 1,533,376 | 10.42 (8.59-12.25) | 0.04 |
| Non-Hispanic American Indian or Alaska Native | 2012 | 163 | 1,556,684 | 12.63 (10.57-14.69) | 0.05 |
| Non-Hispanic American Indian or Alaska Native | 2013 | 149 | 1,581,241 | 10.18 (8.45-11.91) | 0.04 |
| Non-Hispanic American Indian or Alaska Native | 2014 | 165 | 1,608,821 | 11.20 (9.41-12.99) | 0.05 |
| Non-Hispanic American Indian or Alaska Native | 2015 | 204 | 1,637,629 | 12.70 (10.87-14.54) | 0.06 |
| Non-Hispanic American Indian or Alaska Native | 2016 | 195 | 1,667,114 | 12.55 (10.71-14.39) | 0.06 |
| Non-Hispanic American Indian or Alaska Native | 2017 | 214 | 1,695,440 | 13.07 (11.25-14.89) | 0.06 |
| Non-Hispanic American Indian or Alaska Native | 2018 | 220 | 1,722,458 | 12.82 (11.07-14.58) | 0.06 |
| Non-Hispanic American Indian or Alaska Native | 2019 | 207 | 1,746,677 | 11.58 (9.95-13.21) | 0.06 |
| Non-Hispanic American Indian or Alaska Native | 2020 | 235 | 1,771,292 | 12.79 (11.10-14.47) | 0.07 |
| **Non-Hispanic American Indian or Alaska Native** | **Total** | **2,917** | **33,081,153** | **10.30 (9.90-10.69)** | **0.85** |
| Non-Hispanic White | 1999 | 2,533 | 134,935,890 | 1.74 (1.67-1.81) | 0.74 |
| Non-Hispanic White | 2000 | 3,038 | 135,202,971 | 2.07 (2.00-2.15) | 0.88 |
| Non-Hispanic White | 2001 | 2,942 | 135,651,679 | 2.01 (1.94-2.08) | 0.86 |
| Non-Hispanic White | 2002 | 3,060 | 136,026,843 | 2.05 (1.98-2.12) | 0.89 |
| Non-Hispanic White | 2003 | 7,677 | 136,415,868 | 5.05 (4.93-5.16) | 2.23 |
| Non-Hispanic White | 2004 | 10,010 | 136,956,601 | 6.47 (6.34-6.59) | 2.91 |
| Non-Hispanic White | 2005 | 12,063 | 137,614,452 | 7.67 (7.54-7.81) | 3.51 |
| Non-Hispanic White | 2006 | 12,435 | 138,363,022 | 7.78 (7.64-7.92) | 3.62 |
| Non-Hispanic White | 2007 | 13,470 | 139,060,265 | 8.32 (8.17-8.46) | 3.92 |
| Non-Hispanic White | 2008 | 15,304 | 139,772,453 | 9.29 (9.14-9.44) | 4.45 |
| Non-Hispanic White | 2009 | 14,463 | 140,451,863 | 8.65 (8.50-8.79) | 4.21 |
| Non-Hispanic White | 2010 | 15,770 | 140,987,505 | 9.32 (9.17-9.47) | 4.59 |
| Non-Hispanic White | 2011 | 16,432 | 141,789,725 | 9.56 (9.42-9.71) | 4.78 |
| Non-Hispanic White | 2012 | 17,808 | 142,424,668 | 10.19 (10.03-10.34) | 5.18 |
| Non-Hispanic White | 2013 | 18,481 | 143,045,234 | 10.36 (10.21-10.52) | 5.38 |
| Non-Hispanic White | 2014 | 18,874 | 143,642,265 | 10.41 (10.26-10.56) | 5.49 |
| Non-Hispanic White | 2015 | 19,584 | 144,347,874 | 10.62 (10.47-10.77) | 5.70 |
| Non-Hispanic White | 2016 | 20,319 | 144,979,180 | 10.85 (10.70-11.00) | 5.91 |
| Non-Hispanic White | 2017 | 20,671 | 145,456,222 | 10.85 (10.70-11.00) | 6.02 |
| Non-Hispanic White | 2018 | 20,925 | 145,801,376 | 10.87 (10.72-11.03) | 6.09 |
| Non-Hispanic White | 2019 | 20,551 | 146,079,678 | 10.50 (10.35-10.65) | 5.98 |
| Non-Hispanic White | 2020 | 21,507 | 146,337,256 | 10.90 (10.75-11.05) | 6.26 |
| **Non-Hispanic White** | **Total** | **307,917** | **3,095,342,890** | **8.21 (8.18-8.24)** | **89.63** |
| Non-Hispanic Black or African American | 1999 | 236 | 19,808,077 | 1.43 (1.25-1.62) | 0.07 |
| Non-Hispanic Black or African American | 2000 | 245 | 20,058,273 | 1.49 (1.30-1.67) | 0.07 |
| Non-Hispanic Black or African American | 2001 | 278 | 20,404,945 | 1.63 (1.43-1.82) | 0.08 |
| Non-Hispanic Black or African American | 2002 | 241 | 20,695,836 | 1.36 (1.19-1.53) | 0.07 |
| Non-Hispanic Black or African American | 2003 | 618 | 20,982,115 | 3.61 (3.32-3.91) | 0.18 |
| Non-Hispanic Black or African American | 2004 | 758 | 21,327,519 | 4.35 (4.03-4.66) | 0.22 |
| Non-Hispanic Black or African American | 2005 | 998 | 21,703,691 | 5.50 (5.15-5.85) | 0.29 |
| Non-Hispanic Black or African American | 2006 | 1,033 | 22,092,072 | 5.52 (5.17-5.86) | 0.30 |
| Non-Hispanic Black or African American | 2007 | 1,071 | 22,474,128 | 5.67 (5.32-6.02) | 0.31 |
| Non-Hispanic Black or African American | 2008 | 1,321 | 22,857,307 | 6.75 (6.38-7.13) | 0.38 |
| Non-Hispanic Black or African American | 2009 | 1,268 | 23,236,715 | 6.31 (5.95-6.67) | 0.37 |
| Non-Hispanic Black or African American | 2010 | 1,362 | 23,537,629 | 6.65 (6.28-7.01) | 0.40 |
| Non-Hispanic Black or African American | 2011 | 1,471 | 23,958,352 | 6.84 (6.48-7.21) | 0.43 |
| Non-Hispanic Black or African American | 2012 | 1,603 | 24,345,607 | 7.21 (6.84-7.57) | 0.47 |
| Non-Hispanic Black or African American | 2013 | 1,734 | 24,743,381 | 7.49 (7.12-7.85) | 0.50 |
| Non-Hispanic Black or African American | 2014 | 1,767 | 25,244,585 | 7.39 (7.03-7.74) | 0.51 |
| Non-Hispanic Black or African American | 2015 | 1,969 | 25,752,287 | 8.02 (7.65-8.39) | 0.57 |
| Non-Hispanic Black or African American | 2016 | 2,134 | 26,212,105 | 8.27 (7.91-8.64) | 0.62 |
| Non-Hispanic Black or African American | 2017 | 2,168 | 26,762,620 | 8.15 (7.80-8.51) | 0.63 |
| Non-Hispanic Black or African American | 2018 | 2,237 | 27,178,229 | 8.38 (8.03-8.74) | 0.65 |
| Non-Hispanic Black or African American | 2019 | 2,288 | 27,592,123 | 8.25 (7.90-8.60) | 0.67 |
| Non-Hispanic Black or African American | 2020 | 2,566 | 27,969,928 | 9.06 (8.70-9.43) | 0.75 |
| **Non-Hispanic Black or African American** | **Total** | **29,366** | **518,937,524** | **6.27 (6.20-6.35)** | **8.55** |
| Hispanic or Latino | 1999 | 76 | 17,503,631 | 0.76 (0.59-0.96) | 0.60 |
| Hispanic or Latino | 2000 | 97 | 18,219,679 | 0.91 (0.73-1.12) | 0.76 |
| Hispanic or Latino | 2001 | 83 | 19,290,018 | 0.77 (0.61-0.96) | 0.65 |
| Hispanic or Latino | 2002 | 85 | 20,159,630 | 0.73 (0.58-0.91) | 0.67 |
| Hispanic or Latino | 2003 | 377 | 21,011,656 | 3.28 (2.94-3.63) | 2.96 |
| Hispanic or Latino | 2004 | 402 | 21,877,214 | 3.25 (2.92-3.58) | 3.16 |
| Hispanic or Latino | 2005 | 518 | 22,804,023 | 3.98 (3.62-4.34) | 4.07 |
| Hispanic or Latino | 2006 | 507 | 23,743,864 | 3.64 (3.31-3.97) | 3.98 |
| Hispanic or Latino | 2007 | 524 | 24,673,919 | 3.58 (3.26-3.91) | 4.11 |
| Hispanic or Latino | 2008 | 585 | 25,602,850 | 3.83 (3.50-4.16) | 4.59 |
| Hispanic or Latino | 2009 | 549 | 26,504,021 | 3.41 (3.11-3.71) | 4.31 |
| Hispanic or Latino | 2010 | 566 | 27,192,663 | 3.42 (3.13-3.72) | 4.44 |
| Hispanic or Latino | 2011 | 616 | 28,255,675 | 3.46 (3.17-3.74) | 4.84 |
| Hispanic or Latino | 2012 | 698 | 28,988,437 | 3.62 (3.34-3.91) | 5.48 |
| Hispanic or Latino | 2013 | 717 | 29,784,174 | 3.63 (3.35-3.91) | 5.63 |
| Hispanic or Latino | 2014 | 757 | 30,809,714 | 3.62 (3.35-3.89) | 5.94 |
| Hispanic or Latino | 2015 | 884 | 31,761,872 | 3.99 (3.71-4.26) | 6.94 |
| Hispanic or Latino | 2016 | 822 | 32,438,262 | 3.51 (3.26-3.76) | 6.45 |
| Hispanic or Latino | 2017 | 922 | 33,594,503 | 3.80 (3.55-4.06) | 7.24 |
| Hispanic or Latino | 2018 | 847 | 34,350,362 | 3.36 (3.12-3.59) | 6.65 |
| Hispanic or Latino | 2019 | 998 | 35,025,850 | 3.82 (3.57-4.06) | 7.84 |
| Hispanic or Latino | 2020 | 1,106 | 35,758,193 | 4.02 (3.77-4.26) | 8.68 |
| **Hispanic or Latino** | **Total** | **12,736** | **589,350,210** | **3.33 (3.27-3.39)** | **100.00** |
| Non-Hispanic Asian or Pacific Islander | 1999 | 20 | 6,917,321 | 0.39 (0.23-0.62) | 0.01 |
| Non-Hispanic Asian or Pacific Islander | 2000 | 26 | 7,210,256 | 0.49 (0.31-0.73) | 0.01 |
| Non-Hispanic Asian or Pacific Islander | 2001 | 20 | 7,651,274 | 0.37 (0.22-0.59) | 0.01 |
| Non-Hispanic Asian or Pacific Islander | 2002 | 25 | 7,999,855 | 0.42 (0.26-0.63) | 0.01 |
| Non-Hispanic Asian or Pacific Islander | 2003 | 52 | 8,336,941 | 0.85 (0.62-1.13) | 0.02 |
| Non-Hispanic Asian or Pacific Islander | 2004 | 66 | 8,679,634 | 1.13 (0.86-1.45) | 0.02 |
| Non-Hispanic Asian or Pacific Islander | 2005 | 92 | 9,041,913 | 1.43 (1.15-1.77) | 0.03 |
| Non-Hispanic Asian or Pacific Islander | 2006 | 99 | 9,409,587 | 1.46 (1.18-1.79) | 0.03 |
| Non-Hispanic Asian or Pacific Islander | 2007 | 151 | 9,760,888 | 2.07 (1.73-2.41) | 0.04 |
| Non-Hispanic Asian or Pacific Islander | 2008 | 149 | 10,102,040 | 2.04 (1.70-2.38) | 0.04 |
| Non-Hispanic Asian or Pacific Islander | 2009 | 169 | 10,429,408 | 2.06 (1.74-2.38) | 0.05 |
| Non-Hispanic Asian or Pacific Islander | 2010 | 167 | 10,670,562 | 2.03 (1.71-2.34) | 0.05 |
| Non-Hispanic Asian or Pacific Islander | 2011 | 172 | 11,055,808 | 1.91 (1.61-2.20) | 0.05 |
| Non-Hispanic Asian or Pacific Islander | 2012 | 178 | 11,510,641 | 1.93 (1.63-2.22) | 0.05 |
| Non-Hispanic Asian or Pacific Islander | 2013 | 215 | 11,931,284 | 2.07 (1.79-2.36) | 0.06 |
| Non-Hispanic Asian or Pacific Islander | 2014 | 213 | 12,503,895 | 2.02 (1.74-2.29) | 0.06 |
| Non-Hispanic Asian or Pacific Islander | 2015 | 225 | 13,054,155 | 1.94 (1.68-2.20) | 0.07 |
| Non-Hispanic Asian or Pacific Islander | 2016 | 250 | 13,344,756 | 2.13 (1.86-2.40) | 0.07 |
| Non-Hispanic Asian or Pacific Islander | 2017 | 262 | 13,938,546 | 2.06 (1.80-2.31) | 0.08 |
| Non-Hispanic Asian or Pacific Islander | 2018 | 279 | 14,258,765 | 2.11 (1.86-2.36) | 0.08 |
| Non-Hispanic Asian or Pacific Islander | 2019 | 258 | 14,536,839 | 1.89 (1.65-2.12) | 0.08 |
| Non-Hispanic Asian or Pacific Islander | 2020 | 267 | 14,798,344 | 1.86 (1.64-2.09) | 0.08 |
| **Non-Hispanic Asian or Pacific Islander** | **Total** | **3,355** | **237,142,712** | **1.73 (1.67-1.80)** | **0.98** |

## **Supplemental Table S5. U.S. Census region-specific annual AMI-related mortality with co-listed nicotine dependence, 1999-2020.**

| **Stratum** | **Year** | **Deaths** | **Population** | **AAMR (95% CI)** | **% total deaths** |
| --- | --- | --- | --- | --- | --- |
| Northeast | 1999 | 366 | 35,633,134 | 1.00 (0.90-1.10) | 0.10 |
| Northeast | 2000 | 487 | 35,788,687 | 1.31 (1.19-1.43) | 0.14 |
| Northeast | 2001 | 438 | 36,006,250 | 1.16 (1.05-1.27) | 0.12 |
| Northeast | 2002 | 474 | 36,185,082 | 1.24 (1.13-1.35) | 0.13 |
| Northeast | 2003 | 1,709 | 36,346,948 | 4.43 (4.22-4.64) | 0.48 |
| Northeast | 2004 | 2,624 | 36,462,699 | 6.75 (6.49-7.01) | 0.73 |
| Northeast | 2005 | 2,894 | 36,559,788 | 7.34 (7.08-7.61) | 0.81 |
| Northeast | 2006 | 3,862 | 36,682,176 | 9.74 (9.43-10.05) | 1.08 |
| Northeast | 2007 | 3,924 | 36,846,338 | 9.72 (9.41-10.03) | 1.10 |
| Northeast | 2008 | 3,926 | 37,084,149 | 9.58 (9.28-9.89) | 1.10 |
| Northeast | 2009 | 3,617 | 37,339,597 | 8.73 (8.44-9.01) | 1.01 |
| Northeast | 2010 | 3,556 | 37,543,347 | 8.48 (8.20-8.76) | 1.00 |
| Northeast | 2011 | 3,417 | 37,864,117 | 7.98 (7.71-8.25) | 0.96 |
| Northeast | 2012 | 3,657 | 38,158,527 | 8.40 (8.13-8.68) | 1.02 |
| Northeast | 2013 | 3,907 | 38,437,194 | 8.86 (8.58-9.14) | 1.09 |
| Northeast | 2014 | 3,831 | 38,710,627 | 8.48 (8.21-8.75) | 1.07 |
| Northeast | 2015 | 3,901 | 38,965,872 | 8.55 (8.27-8.82) | 1.09 |
| Northeast | 2016 | 4,004 | 39,040,202 | 8.61 (8.34-8.89) | 1.12 |
| Northeast | 2017 | 3,856 | 39,417,175 | 8.10 (7.84-8.36) | 1.08 |
| Northeast | 2018 | 3,907 | 39,321,978 | 8.07 (7.81-8.33) | 1.09 |
| Northeast | 2019 | 3,795 | 39,381,333 | 7.75 (7.50-8.00) | 1.06 |
| Northeast | 2020 | 4,074 | 39,418,559 | 8.28 (8.02-8.54) | 1.14 |
| **Northeast** | **Total** | **66,226** | **827,193,779** | **7.09 (7.03-7.14)** | **18.54** |
| Midwest | 1999 | 698 | 41,293,967 | 1.69 (1.57-1.82) | 0.20 |
| Midwest | 2000 | 842 | 41,504,992 | 2.00 (1.87-2.14) | 0.24 |
| Midwest | 2001 | 847 | 41,762,990 | 1.99 (1.85-2.12) | 0.24 |
| Midwest | 2002 | 853 | 41,970,731 | 1.98 (1.85-2.12) | 0.24 |
| Midwest | 2003 | 1,626 | 42,200,881 | 3.73 (3.55-3.91) | 0.46 |
| Midwest | 2004 | 2,541 | 42,455,980 | 5.72 (5.50-5.95) | 0.71 |
| Midwest | 2005 | 3,094 | 42,748,579 | 6.86 (6.62-7.10) | 0.87 |
| Midwest | 2006 | 2,968 | 43,084,311 | 6.51 (6.27-6.74) | 0.83 |
| Midwest | 2007 | 3,967 | 43,424,366 | 8.52 (8.25-8.79) | 1.11 |
| Midwest | 2008 | 5,586 | 43,718,509 | 11.81 (11.50-12.13) | 1.56 |
| Midwest | 2009 | 5,257 | 44,010,460 | 10.97 (10.67-11.27) | 1.47 |
| Midwest | 2010 | 5,812 | 44,248,465 | 11.93 (11.62-12.24) | 1.63 |
| Midwest | 2011 | 6,615 | 44,584,105 | 13.35 (13.03-13.68) | 1.85 |
| Midwest | 2012 | 6,857 | 44,817,227 | 13.60 (13.27-13.93) | 1.92 |
| Midwest | 2013 | 7,048 | 45,090,597 | 13.69 (13.36-14.01) | 1.97 |
| Midwest | 2014 | 7,118 | 45,360,409 | 13.54 (13.22-13.86) | 1.99 |
| Midwest | 2015 | 6,847 | 45,628,315 | 12.79 (12.49-13.10) | 1.92 |
| Midwest | 2016 | 6,910 | 45,802,491 | 12.77 (12.46-13.08) | 1.93 |
| Midwest | 2017 | 7,180 | 46,143,783 | 13.01 (12.70-13.31) | 2.01 |
| Midwest | 2018 | 7,295 | 46,405,110 | 13.01 (12.70-13.31) | 2.04 |
| Midwest | 2019 | 7,154 | 46,589,364 | 12.51 (12.21-12.80) | 2.00 |
| Midwest | 2020 | 7,427 | 46,721,679 | 12.86 (12.56-13.16) | 2.08 |
| **Midwest** | **Total** | **104,542** | **969,567,311** | **9.67 (9.61-9.73)** | **29.27** |
| South | 1999 | 1,226 | 64,108,630 | 1.93 (1.82-2.04) | 0.34 |
| South | 2000 | 1,422 | 64,843,390 | 2.23 (2.11-2.34) | 0.40 |
| South | 2001 | 1,372 | 65,874,012 | 2.08 (1.97-2.19) | 0.38 |
| South | 2002 | 1,404 | 66,758,178 | 2.09 (1.98-2.20) | 0.39 |
| South | 2003 | 3,942 | 67,639,133 | 5.83 (5.64-6.01) | 1.10 |
| South | 2004 | 3,960 | 68,718,205 | 5.71 (5.53-5.89) | 1.11 |
| South | 2005 | 5,609 | 69,951,038 | 7.91 (7.71-8.12) | 1.57 |
| South | 2006 | 5,059 | 71,198,336 | 6.97 (6.78-7.16) | 1.42 |
| South | 2007 | 5,066 | 72,387,467 | 6.82 (6.63-7.01) | 1.42 |
| South | 2008 | 5,509 | 73,529,724 | 7.18 (6.99-7.37) | 1.54 |
| South | 2009 | 5,293 | 74,596,130 | 6.74 (6.56-6.92) | 1.48 |
| South | 2010 | 5,994 | 75,419,767 | 7.48 (7.29-7.68) | 1.68 |
| South | 2011 | 6,167 | 76,650,163 | 7.48 (7.29-7.66) | 1.73 |
| South | 2012 | 7,158 | 77,681,893 | 8.44 (8.24-8.64) | 2.00 |
| South | 2013 | 7,421 | 78,693,557 | 8.50 (8.30-8.70) | 2.08 |
| South | 2014 | 7,940 | 79,945,474 | 8.86 (8.66-9.05) | 2.22 |
| South | 2015 | 8,990 | 81,260,812 | 9.77 (9.57-9.98) | 2.52 |
| South | 2016 | 9,555 | 82,405,493 | 10.09 (9.88-10.29) | 2.68 |
| South | 2017 | 9,779 | 83,659,214 | 10.07 (9.86-10.27) | 2.74 |
| South | 2018 | 10,100 | 84,717,024 | 10.22 (10.02-10.42) | 2.83 |
| South | 2019 | 10,094 | 85,606,773 | 9.96 (9.76-10.15) | 2.83 |
| South | 2020 | 10,796 | 86,611,804 | 10.41 (10.21-10.61) | 3.02 |
| **South** | **Total** | **133,856** | **1,652,256,217** | **7.50 (7.45-7.54)** | **37.48** |
| West | 1999 | 611 | 39,373,038 | 1.69 (1.55-1.82) | 0.17 |
| West | 2000 | 689 | 39,847,571 | 1.89 (1.75-2.03) | 0.19 |
| West | 2001 | 708 | 40,661,876 | 1.88 (1.74-2.02) | 0.20 |
| West | 2002 | 718 | 41,294,037 | 1.86 (1.72-1.99) | 0.20 |
| West | 2003 | 1,536 | 41,903,467 | 3.88 (3.68-4.07) | 0.43 |
| West | 2004 | 2,229 | 42,568,500 | 5.54 (5.31-5.77) | 0.62 |
| West | 2005 | 2,212 | 43,291,979 | 5.35 (5.12-5.57) | 0.62 |
| West | 2006 | 2,337 | 44,054,536 | 5.53 (5.31-5.76) | 0.65 |
| West | 2007 | 2,424 | 44,745,606 | 5.57 (5.35-5.80) | 0.68 |
| West | 2008 | 2,509 | 45,462,708 | 5.62 (5.40-5.85) | 0.70 |
| West | 2009 | 2,427 | 46,160,829 | 5.27 (5.06-5.49) | 0.68 |
| West | 2010 | 2,674 | 46,680,404 | 5.72 (5.50-5.94) | 0.75 |
| West | 2011 | 2,666 | 47,494,551 | 5.49 (5.28-5.71) | 0.75 |
| West | 2012 | 2,842 | 48,168,390 | 5.65 (5.44-5.86) | 0.80 |
| West | 2013 | 2,970 | 48,863,966 | 5.74 (5.53-5.95) | 0.83 |
| West | 2014 | 2,943 | 49,792,770 | 5.52 (5.32-5.72) | 0.82 |
| West | 2015 | 3,204 | 50,698,818 | 5.86 (5.65-6.07) | 0.90 |
| West | 2016 | 3,323 | 51,393,231 | 5.91 (5.70-6.11) | 0.93 |
| West | 2017 | 3,488 | 52,227,159 | 6.02 (5.82-6.23) | 0.98 |
| West | 2018 | 3,267 | 52,867,078 | 5.53 (5.34-5.72) | 0.91 |
| West | 2019 | 3,319 | 53,403,697 | 5.49 (5.30-5.68) | 0.93 |
| West | 2020 | 3,447 | 53,882,971 | 5.60 (5.41-5.79) | 0.97 |
| **West** | **Total** | **52,543** | **1,024,837,182** | **5.03 (4.98-5.07)** | **14.71** |

## **Supplemental Table S6. State-level AMI-related mortality with co-listed nicotine dependence, 1999-2020.**

| **State** | **Deaths** | **Population** | **AAMR (95% CI)** | **% total deaths** |
| --- | --- | --- | --- | --- |
| North Dakota | 2,227 | 9,822,062 | 19.59 (18.76-20.41) | 0.62 |
| Wyoming | 1,596 | 7,921,067 | 18.63 (17.70-19.55) | 0.45 |
| Idaho | 4,039 | 21,392,071 | 17.37 (16.83-17.91) | 1.13 |
| South Dakota | 2,200 | 11,603,220 | 16.09 (15.41-16.77) | 0.62 |
| Vermont | 1,568 | 9,393,709 | 14.46 (13.74-15.18) | 0.44 |
| Oregon | 8,732 | 57,214,641 | 13.67 (13.38-13.96) | 2.44 |
| Wisconsin | 12,676 | 82,712,057 | 13.54 (13.30-13.78) | 3.55 |
| Arkansas | 6,349 | 41,711,420 | 13.24 (12.92-13.57) | 1.78 |
| Kentucky | 8,556 | 63,061,451 | 12.31 (12.04-12.57) | 2.40 |
| Washington | 12,027 | 98,931,323 | 11.81 (11.60-12.03) | 3.37 |
| Michigan | 18,247 | 145,550,610 | 11.22 (11.05-11.38) | 5.11 |
| Ohio | 21,031 | 169,651,360 | 10.91 (10.76-11.06) | 5.89 |
| Montana | 1,868 | 14,577,633 | 10.85 (10.35-11.35) | 0.52 |
| Indiana | 10,893 | 92,386,904 | 10.72 (10.51-10.92) | 3.05 |
| Rhode Island | 1,941 | 15,765,032 | 10.54 (10.06-11.01) | 0.54 |
| Texas | 33,384 | 344,632,461 | 10.28 (10.17-10.39) | 9.35 |
| New Hampshire | 2,230 | 19,856,602 | 10.16 (9.74-10.59) | 0.62 |
| Oklahoma | 5,948 | 53,110,132 | 10.13 (9.87-10.39) | 1.67 |
| Missouri | 9,879 | 86,518,061 | 10.11 (9.91-10.31) | 2.77 |
| Maine | 2,361 | 20,521,512 | 9.46 (9.07-9.84) | 0.66 |
| Kansas | 4,231 | 39,931,570 | 9.42 (9.13-9.70) | 1.18 |
| Tennessee | 9,053 | 92,689,763 | 8.88 (8.70-9.06) | 2.53 |
| South Carolina | 6,649 | 67,227,226 | 8.85 (8.63-9.06) | 1.86 |
| Maryland | 7,627 | 84,902,583 | 8.73 (8.53-8.93) | 2.14 |
| New Jersey | 12,333 | 130,947,870 | 8.63 (8.48-8.78) | 3.45 |
| Iowa | 4,490 | 44,030,204 | 8.54 (8.29-8.79) | 1.26 |
| Nebraska | 2,478 | 25,869,503 | 8.48 (8.14-8.82) | 0.69 |
| Delaware | 1,214 | 13,210,778 | 8.06 (7.61-8.52) | 0.34 |
| Louisiana | 5,467 | 64,895,449 | 7.90 (7.69-8.11) | 1.53 |
| Pennsylvania | 17,774 | 189,975,429 | 7.81 (7.69-7.92) | 4.98 |
| New Mexico | 2,418 | 28,568,524 | 7.62 (7.31-7.92) | 0.68 |
| North Carolina | 10,778 | 137,233,223 | 7.24 (7.10-7.38) | 3.02 |
| New York | 22,363 | 288,624,005 | 7.12 (7.03-7.21) | 6.26 |
| Utah | 2,040 | 34,057,911 | 6.57 (6.28-6.85) | 0.57 |
| Illinois | 11,968 | 184,489,320 | 6.11 (6.00-6.22) | 3.35 |
| Florida | 21,665 | 289,701,852 | 5.95 (5.87-6.03) | 6.07 |
| Colorado | 4,111 | 73,189,174 | 5.87 (5.69-6.05) | 1.15 |
| Arizona | 5,778 | 90,089,006 | 5.66 (5.52-5.81) | 1.62 |
| Alaska | 408 | 9,623,822 | 5.44 (4.87-6.00) | 0.11 |
| Hawaii | 1,213 | 20,066,183 | 5.32 (5.02-5.62) | 0.34 |
| Minnesota | 4,222 | 77,002,440 | 5.07 (4.91-5.22) | 1.18 |
| Connecticut | 2,985 | 53,012,824 | 4.90 (4.73-5.08) | 0.84 |
| Georgia | 6,331 | 135,415,629 | 4.70 (4.59-4.82) | 1.77 |
| West Virginia | 1,592 | 28,023,276 | 4.70 (4.46-4.93) | 0.45 |
| Mississippi | 1,977 | 41,496,865 | 4.30 (4.10-4.49) | 0.55 |
| Virginia | 4,917 | 116,579,933 | 4.06 (3.94-4.17) | 1.38 |
| Nevada | 1,395 | 38,580,189 | 3.60 (3.41-3.80) | 0.39 |
| District of Columbia | 291 | 9,448,827 | 3.41 (3.01-3.81) | 0.08 |
| Alabama | 2,058 | 68,915,349 | 2.65 (2.53-2.76) | 0.58 |
| Massachusetts | 2,671 | 99,096,796 | 2.39 (2.30-2.48) | 0.75 |
| California | 6,918 | 530,625,638 | 1.29 (1.26-1.32) | 1.94 |

## **Supplemental Table S7. Urbanization-specific annual AMI-related mortality with co-listed nicotine dependence, 1999-2020.**

| **Stratum** | **Year** | **Deaths** | **Population** | **CMR (95% CI)** | **AAMR (95% CI)** | **% total deaths** |
| --- | --- | --- | --- | --- | --- | --- |
| Metropolitan | 1999 | 2,061 | 151,245,342 | 1.36 (1.30-1.42) | 1.41 (1.35-1.47) | 0.80 |
| Metropolitan | 2000 | 2,421 | 152,658,699 | 1.59 (1.52-1.65) | 1.65 (1.58-1.72) | 0.94 |
| Metropolitan | 2001 | 2,353 | 154,896,258 | 1.52 (1.46-1.58) | 1.54 (1.48-1.60) | 0.92 |
| Metropolitan | 2002 | 2,456 | 156,659,051 | 1.57 (1.51-1.63) | 1.60 (1.54-1.67) | 0.96 |
| Metropolitan | 2003 | 6,414 | 158,367,715 | 4.05 (3.95-4.15) | 4.10 (4.00-4.20) | 2.50 |
| Metropolitan | 2004 | 8,236 | 160,272,097 | 5.14 (5.03-5.25) | 5.20 (5.09-5.31) | 3.21 |
| Metropolitan | 2005 | 10,193 | 162,371,826 | 6.28 (6.16-6.40) | 6.31 (6.19-6.44) | 3.97 |
| Metropolitan | 2006 | 10,612 | 164,523,389 | 6.45 (6.33-6.57) | 6.45 (6.33-6.58) | 4.13 |
| Metropolitan | 2007 | 11,567 | 166,650,886 | 6.94 (6.81-7.07) | 6.87 (6.74-7.00) | 4.51 |
| Metropolitan | 2008 | 12,985 | 168,826,027 | 7.69 (7.56-7.82) | 7.54 (7.41-7.67) | 5.06 |
| Metropolitan | 2009 | 12,225 | 170,965,574 | 7.15 (7.02-7.28) | 6.94 (6.82-7.07) | 4.76 |
| Metropolitan | 2010 | 12,987 | 172,591,105 | 7.52 (7.40-7.65) | 7.24 (7.11-7.36) | 5.06 |
| Metropolitan | 2011 | 13,452 | 175,204,532 | 7.68 (7.55-7.81) | 7.30 (7.17-7.42) | 5.24 |
| Metropolitan | 2012 | 14,526 | 177,423,676 | 8.19 (8.05-8.32) | 7.66 (7.53-7.79) | 5.66 |
| Metropolitan | 2013 | 15,238 | 179,634,449 | 8.48 (8.35-8.62) | 7.82 (7.70-7.95) | 5.94 |
| Metropolitan | 2014 | 15,526 | 182,304,016 | 8.52 (8.38-8.65) | 7.77 (7.65-7.90) | 6.05 |
| Metropolitan | 2015 | 16,200 | 184,959,306 | 8.76 (8.62-8.89) | 7.89 (7.76-8.01) | 6.31 |
| Metropolitan | 2016 | 16,860 | 186,963,190 | 9.02 (8.88-9.15) | 8.02 (7.89-8.14) | 6.57 |
| Metropolitan | 2017 | 17,275 | 189,675,062 | 9.11 (8.97-9.24) | 8.01 (7.89-8.13) | 6.73 |
| Metropolitan | 2018 | 17,538 | 191,415,024 | 9.16 (9.03-9.30) | 7.98 (7.86-8.10) | 6.83 |
| Metropolitan | 2019 | 17,300 | 193,006,488 | 8.96 (8.83-9.10) | 7.71 (7.59-7.82) | 6.74 |
| Metropolitan | 2020 | 18,296 | 194,600,110 | 9.40 (9.27-9.54) | 8.01 (7.89-8.13) | 7.13 |
| **Metropolitan** | **Total** | **256,721** | **3,795,213,822** | **6.76 (6.74-6.79)** | **6.43 (6.40-6.45)** | **100.00** |
| Nonmetropolitan | 1999 | 840 | 29,163,427 | 2.88 (2.69-3.08) | 2.61 (2.43-2.79) | 0.84 |
| Nonmetropolitan | 2000 | 1,019 | 29,325,941 | 3.47 (3.26-3.69) | 3.15 (2.95-3.34) | 1.01 |
| Nonmetropolitan | 2001 | 1,012 | 29,408,870 | 3.44 (3.23-3.65) | 3.06 (2.87-3.25) | 1.01 |
| Nonmetropolitan | 2002 | 993 | 29,548,977 | 3.36 (3.15-3.57) | 2.99 (2.81-3.18) | 0.99 |
| Nonmetropolitan | 2003 | 2,399 | 29,722,714 | 8.07 (7.75-8.39) | 7.03 (6.74-7.31) | 2.39 |
| Nonmetropolitan | 2004 | 3,118 | 29,933,287 | 10.42 (10.05-10.78) | 9.02 (8.70-9.34) | 3.10 |
| Nonmetropolitan | 2005 | 3,616 | 30,179,558 | 11.98 (11.59-12.37) | 10.31 (9.97-10.65) | 3.60 |
| Nonmetropolitan | 2006 | 3,614 | 30,495,970 | 11.85 (11.46-12.24) | 10.12 (9.79-10.46) | 3.60 |
| Nonmetropolitan | 2007 | 3,814 | 30,752,891 | 12.40 (12.01-12.80) | 10.52 (10.18-10.85) | 3.80 |
| Nonmetropolitan | 2008 | 4,545 | 30,969,063 | 14.68 (14.25-15.10) | 12.28 (11.92-12.64) | 4.52 |
| Nonmetropolitan | 2009 | 4,369 | 31,141,442 | 14.03 (13.61-14.45) | 11.63 (11.28-11.98) | 4.35 |
| Nonmetropolitan | 2010 | 5,049 | 31,300,878 | 16.13 (15.69-16.58) | 13.34 (12.97-13.72) | 5.03 |
| Nonmetropolitan | 2011 | 5,413 | 31,388,404 | 17.25 (16.79-17.70) | 14.08 (13.70-14.46) | 5.39 |
| Nonmetropolitan | 2012 | 5,988 | 31,402,361 | 19.07 (18.59-19.55) | 15.40 (15.00-15.80) | 5.96 |
| Nonmetropolitan | 2013 | 6,108 | 31,450,865 | 19.42 (18.93-19.91) | 15.42 (15.02-15.81) | 6.08 |
| Nonmetropolitan | 2014 | 6,306 | 31,505,264 | 20.02 (19.52-20.51) | 15.75 (15.35-16.14) | 6.28 |
| Nonmetropolitan | 2015 | 6,742 | 31,594,511 | 21.34 (20.83-21.85) | 16.74 (16.33-17.15) | 6.71 |
| Nonmetropolitan | 2016 | 6,932 | 31,678,227 | 21.88 (21.37-22.40) | 16.83 (16.42-17.24) | 6.90 |
| Nonmetropolitan | 2017 | 7,028 | 31,772,269 | 22.12 (21.60-22.64) | 16.86 (16.46-17.27) | 7.00 |
| Nonmetropolitan | 2018 | 7,031 | 31,896,166 | 22.04 (21.53-22.56) | 16.69 (16.28-17.09) | 7.00 |
| Nonmetropolitan | 2019 | 7,062 | 31,974,679 | 22.09 (21.57-22.60) | 16.50 (16.10-16.89) | 7.03 |
| Nonmetropolitan | 2020 | 7,448 | 32,028,405 | 23.25 (22.73-23.78) | 17.34 (16.93-17.75) | 7.41 |
| **Nonmetropolitan** | **Total** | **100,446** | **678,634,169** | **14.80 (14.71-14.89)** | **12.09 (12.02-12.17)** | **100.00** |
